# The Antiviral Efficacy and Safety of Azvudine in Hospitalized SARS‐CoV‐2 Infected Patients with Liver Diseases Based on a Multicenter, Retrospective Cohort Study

**DOI:** 10.1002/advs.202405679

**Published:** 2025-02-22

**Authors:** Junyi Sun, Mengzhao Yang, Guanyue Su, Ling Wang, Xiaobo Hu, Yongjian Zhou, Guangying Cui, Guowu Qian, Yiqiang Yuan, Xinjun Hu, Silin Li, Hong Luo, Shixi Zhang, Guangming Li, Donghua Zhang, Guotao Li, Ming Cheng, Zujiang Yu, Zhigang Ren

**Affiliations:** ^1^ Department of Infectious Diseases State Key Laboratory of Antiviral Drugs Pingyuan Laboratory The First Affiliated Hospital of Zhengzhou University Zhengzhou 450052 China; ^2^ Department of Cardiovascular Medicine Henan Provincial Chest Hospital Affiliated to Zhengzhou University Zhengzhou 450008 China; ^3^ Department of Gastrointestinal Surgery Nanyang Central Hospital Nanyang 473009 China; ^4^ Department of Infectious Diseases The First Affiliated Hospital College of Clinical Medicine Henan University of Science and Technology Luoyang 471003 China; ^5^ Department of Respiratory and Critical Care Medicine Fengqiu County People's Hospital Xinxiang 453300 China; ^6^ Guangshan County People's Hospital Guangshan County Xinyang 465450 China; ^7^ Department of Infectious Diseases Shangqiu Municipal Hospital Shangqiu 476000 China; ^8^ Department of Liver Disease The Affiliated Infectious Disease Hospital of Zhengzhou University Zhengzhou 450052 China; ^9^ Department of Infectious Diseases Anyang City Fifth People's Hospital Anyang 455000 China; ^10^ Department of Infectious Diseases Luoyang Central Hospital Affiliated to Zhengzhou University Luoyang 471000 China; ^11^ Department of Medical Information The First Affiliated Hospital of Zhengzhou University Zhengzhou 450052 China

**Keywords:** all‐cause death, azvudine, composite disease progression, real‐world, SARS‐CoV‐2

## Abstract

Despite azvudine being prioritized for the treatment of severe acute respiratory syndrome coronavirus 2 (SARS‐CoV‐2) infection, its effectiveness and safety remain inadequately substantiated in hospitalized SARS‐CoV‐2 infected patients with liver diseases. A retrospective nine‐center cohort study along with an independent validation cohort is conducted to examine the efficacy of azvudine (Clinical Trial Registration Number: NCT06349655). The primary outcome is all‐cause mortality and the secondary outcome is composite disease progression. Efficacy is assessed via Kaplan–Meier analysis and Cox regression, with subgroup and sensitivity analyses for further validation. Among 32 864 hospitalized SARS‐CoV‐2 infected patients, 1022 eligible azvudine recipients, and 1022 controls are included through propensity score match. Kaplan–Meier analysis reveals that azvudine treatment is associated with a lower risk of all‐cause mortality and composite disease progression (both *p*<0.0001). Cox regression analysis suggests azvudine recipients could have a 39% lower risk of all‐cause mortality than controls (95% confidence interval [CI]: 0.468–0.795, *p*<0.001), but with no notable significance in composite disease progression (hazard ratio: 0.85, 95% CI: 0.686‐1.061, *p* = 0.154). Subgroup analysis suggests that azvudine has a greater benefit for both all‐cause mortality and composite disease progression in patients with kidney diseases or without autoimmune diseases. Three sensitivity analyses and validation cohorts confirm the robustness of the findings. Safety analysis observes few adverse events in azvudine recipients. Within 15 days after azvudine administration, no significant difference in liver function indexes and kidney function indexes is observed between the two groups except for a few time points. These findings demonstrate that azvudine shows potential clinical efficacy in improving all‐cause mortality in hospitalized SARS‐CoV‐2 infected patients with liver diseases, with acceptable adverse effects.

## Graphical abstract

Azvudine shows benefits in reducing all‐cause mortality in hospitalized SARS‐CoV‐2 infected patients and liver diseases. COVID‐19 patients with kidney disease and without autoimmune diseases show a preference for azvudine. Azvudine may be a promising antiviral agent for treating COVID‐19 concomitant with liver diseases.

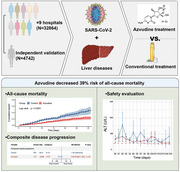

## 1 Introduction

According to the reports of the World Health Organization (WHO), the cumulative number of Coronavirus Disease 2019 (COVID‐19) cases has exceeded 777 million. Data showed that in the past few years, the weekly increase in the number of COVID‐19 cases generally did not exceed 10 million. However, during the fourth week of December 2022, the number of COVID‐19 infections exceeded 40 million, which represented the largest number of infections in a short period since the outbreak of COVID‐19 (WHO COVID‐19 dashboard. Number of COVID‐19 cases reported to WHO.^[^ ^1^ ^]^ The global spread of the COVID‐19 pandemic has posed significant challenges for patients with concurrent liver diseases.^[^ ^2^, ^3^ ^]^ As a crucial metabolic and immunoregulatory organ, the liver may be directly affected by Severe Acute Respiratory Syndrome Coronavirus 2 (SARS‐CoV‐2) infection.^[^ ^4^, ^5^ ^]^ It has been reported that COVID‐19 patients with underlying liver diseases are more prone to severe complications and adverse outcomes compared to those without liver diseases.^[^ ^6^, ^7^, ^8^, ^9^, ^10^ ^]^ Numerous studies have investigated the clinical characteristics of SARS‐CoV‐2 infection as well as the immune responses induced by infection or vaccination.^[^ ^11^, ^12^, ^13^, ^14^, ^15^ ^]^ However, most of these studies focused on healthy individuals. Consequently, there is limited understanding of the clinical impact of SARS‐CoV‐2 infection and the role of the adaptive immune response in vulnerable populations, including those with liver diseases.

Various therapeutic interventions, including Dexamethasone, Remdesivir, and Paxlovid, have been shown to effectively reduce mortality in symptomatic SARS‐CoV‐2 patients.^[^ ^16^, ^17^, ^18^ ^]^ However, some of these treatments may also result in liver injury. For instance, Remdesivir, a nucleotide analog RNA polymerase inhibitor, has been reported to cause hepatocellular damage.^[^ ^19^ ^]^ Consequently, there is an urgent need to identify effective and safe treatment strategies for managing COVID‐19 in patients with concurrent liver diseases.

Azvudine, as a nucleoside analog reverse transcriptase inhibitor, is known for its broad‐spectrum antiviral activity, particularly against RNA viruses.^[^ ^20^ ^]^ Due to its antiviral properties, azvudine has been included in the COVID‐19 treatment guidelines in China.^[^ ^21^, ^22^, ^23^ ^]^ In addition, azvudine may modulate immune responses and the production of inflammatory mediators, exerting a positive impact on viral infection and related complications. In addition, azvudine was associated with a significantly lower risk of composite disease progression outcomes compared with controls, especially in males and patients with severe COVID‐19.^[^ ^24^ ^]^ Currently, there is no evidence to suggest significant clinical implications of azvudine in COVID‐19 patients with concurrent liver diseases. Therefore, further clinical research is needed to confirm the exact efficacy and safety of azvudine in this population.

This study aimed to investigate the clinical efficacy and safety of azvudine in treating hospitalized patients with SARS‐CoV‐2 infection complicated by liver diseases. Therefore, a multicenter cohort with a large sample size was established by collecting clinical data from nine hospitals in Henan Province, China. Moreover, an independent validation cohort was conducted to validate the efficacy of azvudine. By comparing the clinical outcomes between azvudine and control groups, stronger evidence was provided for the clinical application of azvudine in hospitalized SARS‐CoV‐2 patients with liver diseases.

## 2 Results

### 2.1 Study Population

**Figure 1:**
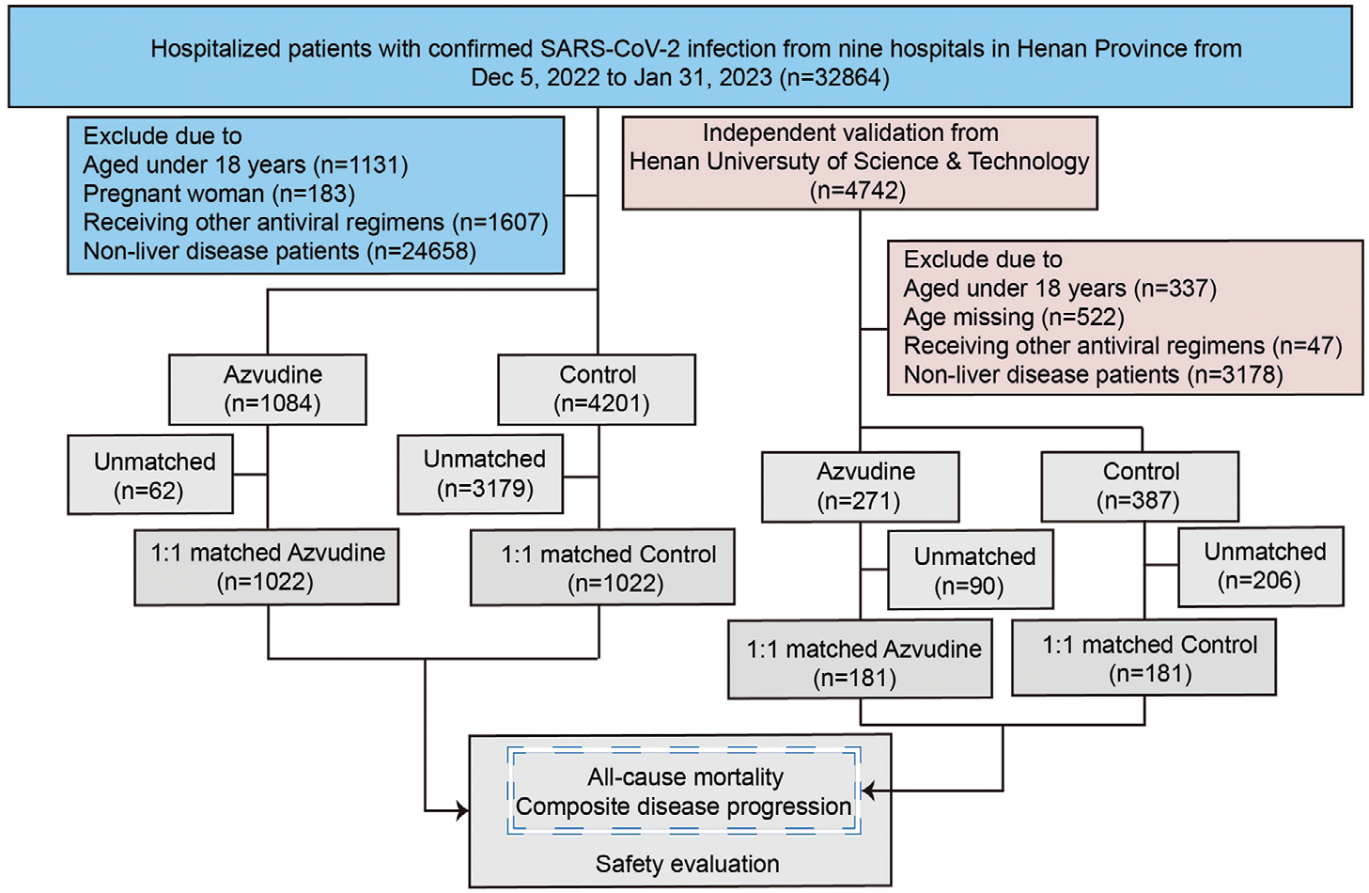
The cohort flow diagram. Study population flowchart showing the inclusion and exclusion of azvudine recipients and their matched controls among hospitalized patients with SARS‐CoV‐2 infection during the study period.

A total of 32 864 hospitalized patients with SARS‐CoV‐2 infection were admitted from nine hospitals in Henan Province. Following the strict inclusion and exclusion criteria, 1084 recipients of azvudine and 4201 recipients of conventional treatment without any antivirals qualified for the azvudine group and control group, respectively. By using propensity score matching to balance the baseline characteristics between the two groups, a 1:1 ratio was confirmed as optimal, resulting in 1022 patients in the control group being enrolled to match 1022 azvudine recipients for final comparison (**Figure** **1**).

The patient baseline characteristics in both the azvudine and control groups before and after propensity score matching were presented in **Table** **1**. Notably, statistical differences were evident across various covariates prior to matching, including age (*p*<0.001), gender (*p* = 0.012), severity at admission (*p*<0.001), concomitant hormone therapy (*p*<0.001), antibiotics (*p*<0.001), most of the comorbidities and laboratory parameters. However, following the 1:1 propensity score matching, the baseline characteristics of all covariates achieved balanced between the azvudine and control groups, with the *P* value >0.05 (Figure S1, Supporting Information).

**Table 1:** Baseline characteristics of the study population before and after 1:1 propensity score matching.

Baseline characteristics	Before matching			After propensity score matching (1:1)		
	Control [*n* = 4201]	Azvudine [*n* = 1084]	*p*‐value	Control [*n* = 1022]	Azvudine [*n* = 1022]	*p‐*value
Sociodemographic information						
Age, years [mean ± SD]	62.57 (14.51)	66.51 (14.34)	<0.001	66.98 (14.31)	66.14 (14.39)	0.184
Gender, n [%]			0.012			0.71
Male	2567 (61.1)	708 (65.3)		675 (66.0)	666 (65.2)	
Female	1634 (38.9)	376 (34.7)		347 (34.0)	356 (34.8)	
Body mass index (BMI), kg/m^2^ [mean ± SD]	24.15 (3.97)	24.36 (4.03)	0.121	24.10 (3.99)	24.34 (4.04)	0.175
Time from diagnosis to treatment exposure, n [%]^a)^			NA			NA
> 5 days	NA	233 (21.5)		NA	226 (22.1)	
≤ 5 days	NA	851 (78.5)		NA	796 (77.9)	
Severity at admission, n [%]			<0.001			0.539
Mild	370 (8.8)	67 (6.2)		56 (5.5)	61 (6.0)	
Moderate	3101 (73.8)	705 (65.0)		654 (64.0)	671 (65.7)	
Severe	730 (17.4)	312 (28.8)		312 (30.5)	290 (28.4)	
Vaccination doses [%]			0.331			0.982
Unvaccinated	1137 (27.1)	327 (30.2)		312 (30.5)	307 (30.0)	
One dose	192 (4.6)	55 (5.1)		53 (5.2)	51 (5.0)	
Two doses	669 (15.9)	162 (14.9)		151 (14.8)	156 (15.3)	
Three doses	2171 (51.7)	530 (48.9)		493 (48.2)	498 (48.7)	
Four doses	30 (0.7)	9 (0.8)		11 (1.1)	9 (0.9)	
Five doses	2 (0.0)	1 (0.1)		2 (0.2)	1 (0.1)	
Concomitant systemic steroid, n [%]			<0.001			1
No	3165 (75.3)	596 (55.0)		591 (57.8)	592 (57.9)	
Yes	1036 (24.7)	488 (45.0)		431 (42.2)	430 (42.1)	
Antibiotics, n [%]			<0.001			0.536
No	2801 (66.7)	521 (48.1)		497 (48.6)	512 (50.1)	
Yes	1400 (33.3)	563 (51.9)		525 (51.4)	510 (49.9)	
Comorbidities, n [%]						
Diabetes			0.861			1
No	3357 (79.9)	863 (79.6)		807 (79.0)	806 (78.9)	
Yes	844 (20.1)	221 (20.4)		215 (21.0)	216 (21.1)	
Hypertension			<0.001			0.722
No	2809 (66.9)	585 (54.0)		572 (56.0)	563 (55.1)	
Yes	1392 (33.1)	499 (46.0)		450 (44.0)	459 (44.9)	
Cardio‐cerebral diseases			<0.001			0.626
No	2818 (67.1)	792 (73.1)		719 (70.4)	730 (71.4)	
Yes	1383 (32.9)	292 (26.9)		303 (29.6)	292 (28.6)	
Kidney diseases			<0.001			0.963
No	3438 (81.8)	642 (59.2)		643 (62.9)	641 (62.7)	
Yes	763 (18.2)	442 (40.8)		379 (37.1)	381 (37.3)	
Primary malignant tumor			<0.001			0.841
No	2877 (68.5)	959 (88.5)		893 (87.4)	897 (87.8)	
Yes	1324 (31.5)	125 (11.5)		129 (12.6)	125 (12.2)	
Chronic respiratory diseases, n [%]			0.062			0.737
No	3491 (83.1)	874 (80.6)		820 (80.2)	827 (80.9)	
Yes	710 (16.9)	210 (19.4)		202 (19.8)	195 (19.1)	
Autoimmune diseases, n [%]			0.541			0.65
No	4010 (95.5)	1040 (95.9)		984 (96.3)	979 (95.8)	
Yes	191 (4.5)	44 (4.1)		38 (3.7)	43 (4.2)	
Laboratory parameters, [mean ± SD]						
Neutrophil, ×10^9^/L	5.79 (4.68)	6.04 (4.06)	0.104	6.31 (4.83)	6.05 (4.09)	0.192
Lymphocyte, ×10^9^/L	1.25 (2.36)	1.05 (1.24)	0.008	1.05 (0.71)	1.07 (1.27)	0.768
Glucose, mmol/L	7.16 (3.85)	7.90 (3.99)	<0.001	7.78 (4.47)	7.84 (3.98)	0.769
High‐density lipoprotein, mmol/L	1.07 (1.79)	1.20 (2.71)	0.074	1.12 (2.15)	1.17 (2.49)	0.642
Low‐density lipoprotein, mmol/L	2.35 (1.72)	2.37 (2.16)	0.688	2.32 (1.04)	2.38 (2.21)	0.411
Alanine aminotransferase, IU/L	64.41 (222.36)	56.65 (105.38)	0.264	62.97 (212.77)	57.51 (108.03)	0.465
Aspartate aminotransferase, IU/L	82.91 (371.25)	56.03 (92.12)	0.018	62.04 (125.07)	56.65 (94.68)	0.272
Creatinine, µmol/L	116.33 (324.81)	100.48 (185.61)	0.123	105.51 (154.36)	101.92 (190.94)	0.64
glomerular filtration rate, mL/min	118.04 (165.32)	111.19 (149.25)	0.216	109.42 (144.61)	110.58 (149.31)	0.858
C–reactive protein, mg/L	50.61 (66.68)	56.82 (69.58)	0.007	57.78 (70.20)	57.53 (70.50)	0.936
Procalcitonin, ng/mL	2.13 (10.80)	1.30 (7.15)	0.016	1.59 (7.59)	1.34 (7.35)	0.438
Prothrombin time, s	15.10 (8.67)	18.60 (10.10)	<0.001	17.76 (10.13)	17.89 (9.50)	0.776
Activated partial thromboplastin time, s	29.46 (9.70)	25.12 (11.24)	<0.001	26.71 (10.75)	25.77 (11.14)	0.051
Cholesterol, mmol/L	4.22 (5.98)	4.24 (6.03)	0.907	4.07 (1.99)	4.27 (6.20)	0.346
Triglyceride, mmol/L	1.76 (4.09)	1.54 (2.38)	0.094	1.50 (1.39)	1.54 (2.43)	0.692
Alkaline phosphatase, IU/L	116.80 (130.29)	96.01 (76.91)	<0.001	100.60 (74.05)	97.27 (78.65)	0.326
Gamma‐glutamyl transpeptidase, IU/L	91.62 (154.68)	85.19 (205.50)	0.256	87.20 (141.37)	86.32 (210.93)	0.911
Albumin, g/L	36.16 (11.21)	34.51 (12.96)	<0.001	34.66 (11.00)	34.67 (13.25)	0.983
Total bilirubin, µmol/L	24.49 (55.32)	15.69 (25.23)	<0.001	17.24 (24.20)	15.85 (25.93)	0.21

^a)^ No values are presented in control groups because there exists no treatment exposure in populations receiving conventional treatment.

### 2.2 Main Outcome: All‐Cause Mortality

**Figure 2:**
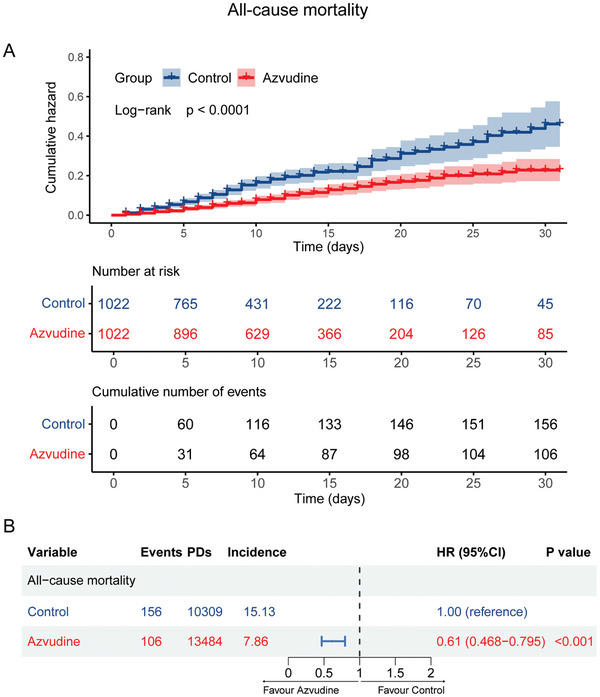
Effectiveness of azvudine versus control in reducing all‐cause mortality for oral treatment of SARS‐CoV‐2 infected patients with pre‐existing liver diseases. A) Kaplan–Meier analysis estimating the cumulative incidence of all‐cause death outcomes; B) Cox regression hazard ratios with 95% confidence intervals.

In a cohort of 2044 patients matched by propensity score, 262 patients (12.82%) died. Among these, 106 deaths (10.37%) occurred in the azvudine group, and 156 deaths (15.26%) occurred in the control group. Kaplan–Meier analysis and log‐rank test results indicated that the 30‐day cumulative all‐cause mortality was significantly lower in the azvudine group compared to the control group (*p*<0.0001) (**Figure** **2**A). After multivariable adjustment using Cox regression analysis, azvudine treatment was significantly associated with a reduced risk of all‐cause mortality compared to the control group (HR: 0.61, 95% confidence interval [CI]: 0.468–0.795, *p*<0.001) (Figure 2B).

**Figure 3:**
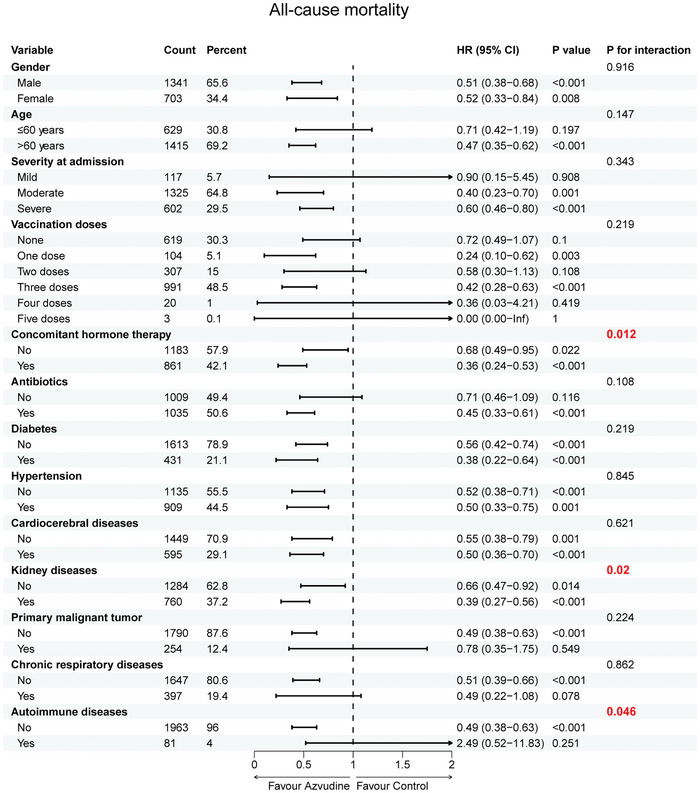
Effectiveness of azvudine in reducing the risk of all‐cause mortality by subgroups of selected baseline characteristics. HR: Hazard Ratio; 95%CI: 95% confidence interval.

In addition, subgroup analysis results showed that the point estimates of HR were generally consistent across subgroups defined by gender, age, severity at admission, antibiotics, and the presence of diabetes, hypertension, cardiovascular diseases, primary malignant tumors, and chronic respiratory diseases. However, significant interaction effects were observed in subgroups that received hormone therapy at the time of diagnosis (*p =* 0.012), whether with concomitant kidney diseases (*p =* 0.02), or whether with concomitant autoimmune diseases. Notably, patients receiving hormone therapy (HR: 0.36; 95% CI: 0.24–0.53), those with kidney disease (HR: 0.39; 95% CI: 0.27–0.56) and those without autoimmune diseases (HR: 0.49; 95% CI: 0.38–0.63) showed a strong preference for azvudine treatment (**Figure** **3**).

To validate the robustness of our findings, sensitivity analyses were conducted. Utilizing propensity score matching with Probit regression (named as Probit regression method, Table S1, Supporting Information), the results indicated a significant decrease in the risk of all‐cause mortality in the azvudine group compared to the control group (log‐rank test: *p =* 0.00013; Cox regression analysis: HR: 0.72, 95% CI: 0.556–0.933, *p =* 0.013) (Figure S2, Supporting Information). Furthermore, missing data were imputed using mean values (named as mean imputation method, Table S2, Supporting Information) and the results also revealed a significant reduction in the cumulative incidence rate of all‐cause mortality in the azvudine group compared to the control group (log‐rank test: *p =* 0.00023; Cox regression analysis: HR: 0.73, 95% CI: 0.556–0.946, *p =* 0.018) (Figure S3, Supporting Information). In addition, another sensitivity analysis was conducted by excluding individuals who were discharged or deceased on the day of medication initiation (named as narrow population method, Table S3, Supporting Information). Kaplan–Meier analysis reiterated a significant reduction in the cumulative incidence rate of all‐cause mortality in the azvudine group compared to the control group (*p*<0.0001) (Figure S4, Supporting Information). Consistent results were demonstrated through Cox regression analysis, suggesting an association between azvudine and reduced all‐cause mortality (HR: 0.71, 95% CI: 0.538–0.932; *p =* 0.014) (Figure S4, Supporting Information).

### 2.3 Secondary Outcome: Composite Disease Progression

**Figure 4:**
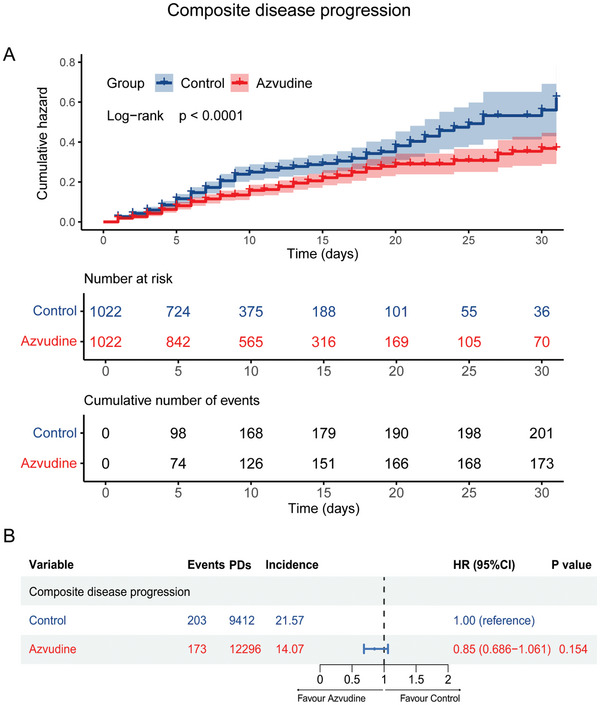
Effectiveness of azvudine versus control in reducing composite disease progression for oral treatment of SARS‐CoV‐2 infected patients with pre‐existing liver diseases. A) Kaplan–Meier analysis estimating the cumulative incidence of composite disease progression outcomes; B) Cox regression hazard ratios with 95% confidence intervals.

Regarding composite disease progression, 376 cases were reported, with 173 cases (16.93%) in the azvudine group and 203 cases (19.86%) in the control group. Kaplan–Meier analysis showed a significant difference in the cumulative incidence of composite disease progression between patients treated with azvudine and the control group (*p*<0.0001) (**Figure** **4**A). Multivariable Cox regression analysis indicated that there was no significant association between azvudine treatment and the control group regarding composite disease progression (HR: 0.85, 95% CI: 0.686–1.061, *p* = 0.154) (Figure 4B).

Further subgroup analysis was performed in populations stratified by interested baseline characteristics, including gender, age, severity at admission, vaccination status, concomitant hormone therapy, antibiotics, and concomitant diseases. Consistent results were observed in most subgroups, while significant interaction effects were observed in subgroups that received vaccination (*p* = 0.039), whether with concomitant kidney diseases (*p* = 0.016), or whether with concomitant autoimmune diseases. Notably, patients receiving vaccination (one dose: HR: 0.31; 95% CI: 0.13–0.74; two doses: HR: 0.49; 95% CI: 0.28–0.85; three doses: HR: 0.61; 95% CI: 0.44–0.84), those with kidney disease (HR: 0.52; 95% CI: 0.39–0.70) and those without autoimmune diseases (HR: 0.64; 95% CI: 0.52–0.79) showed a strong preference for azvudine treatment (Figure S5, Supporting Information).

Three sensitivity analyses were then used to identify the robustness of the results. According to Kaplan–Meier analysis results, azvudine was associated with a lower cumulative risk of composite disease progression in the mean imputation method (*p* = 0.019) (Figure S6, Supporting Information) and narrow population method (*p* = 0.032) (Figure S7, Supporting Information), while no significant difference was observed in Probit regression method (*p* = 0.13) (Figure S8, Supporting Information). Multivariable Cox regression demonstrated no difference between azvudine and the control group regarding composite disease progression in all three sensitivity analysis methods (Figures S6–S8, Supporting Information).

**Figure 5:**
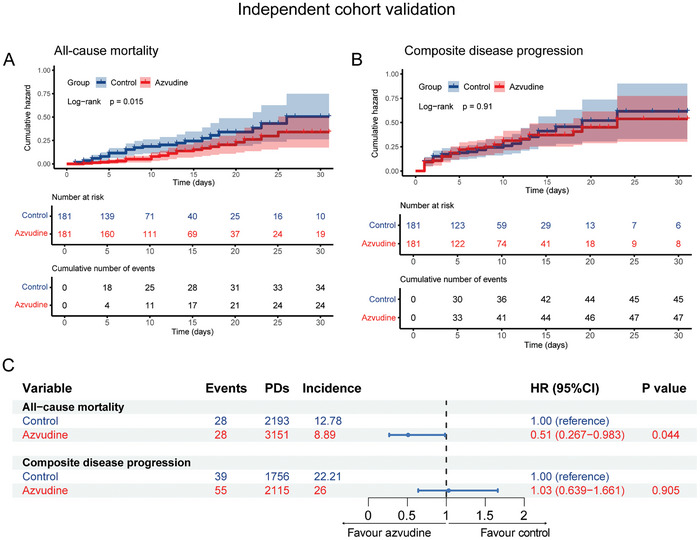
Effectiveness of azvudine versus control in reducing all‐cause mortality and composite disease progression in an independent cohort from the First Affiliated Hospital of Henan University of Science & Technology. A,B) Kaplan–Meier analysis estimating the cumulative incidence of all‐cause mortality (A) and composite disease progression outcomes (B); C) Cox regression hazard ratios with 95% confidence intervals for all‐cause mortality and composite disease progression outcomes one.

### 2.4 An Independent Cohort Validation

To identify the generalization of the results in other cohort, we collected 4742 SARS‐CoV‐2 infected patients from other hospitals, the First Affiliated Hospital of Henan University of Science & Technology (Luoyang, Henan). After strict screening and 1:1 PSM, 181 azvudine recipients and 181 matched controls were enrolled in the cohort (Table S4, Supporting Information). By using the same analysis methods as above, we found similar results that azvudine treatment showed significant effectiveness in reducing all‐cause mortality by either Kaplan–Meier analysis (*p* = 0.015) and Cox regression analysis (HR: 0.53, 95% CI: 0.297–0.928, *p* = 0.026) (**Figure** **5**A,C). However, no significant difference existed between the two groups regarding composite disease progression (log‐rank: *p* = 0.91; HR: 1.10, 95% CI: 0.748–1.609, *p* = 0.637) (Figure **5**B,C).

**Figure 6:**
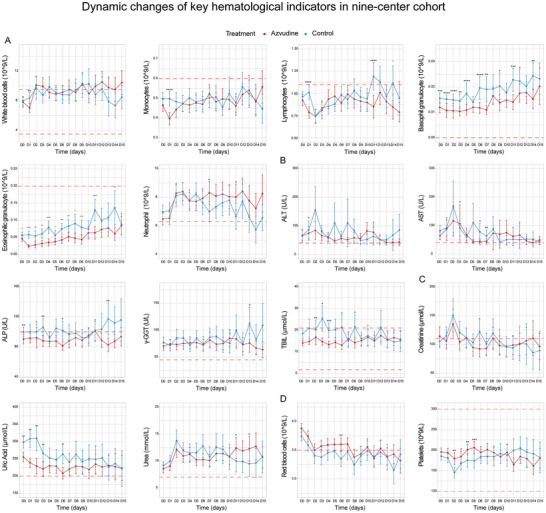
Dynamic changes of key hematological indicators within 15 days after taking azvudine. A) Immune‐related indicators in blood routine examination, including white blood cells, monocytes, lymphocytes, basophilic granulocytes, eosinophilic granulocytes, and neutrophils; B) Liver function‐related indicators, including ALT, AST, ALP, GGT, and TBIL; C) Kidney function‐related indicators, including creatinine, uric acid, and urea; D) Blood clotting function‐related indicators, including red blood cells and platelets.

### 2.5 Safety Evaluation

To assess the safety of azvudine, data on adverse events following azvudine administration were collected and listed in **Table** **2**. During the follow‐up period, azvudine recipients had a higher risk of alanine aminotransferase (ALT) abnormalities (azvudine 34% vs control 28%, *p* = 0.044) and decreased lymphocytes (azvudine 37% vs control 30%, *p* = 0.004). Conversely, compared to the control group, azvudine was associated with a lower risk of serum phosphorus abnormalities (azvudine 14% vs control 23%, *p* = 0.006), and decreased platelets (azvudine 15% vs control 21%, *p* = 0.014). For serious adverse events graded greater than 3, azvudine treatment lowered the occurrence rate of decreased platelets (azvudine 6.2% vs control 10%, *p* = 0.023) while increasing the risk of decreased lymphocytes (azvudine 23% vs control 17%, *p* = 0.001) and increased neutrophils (azvudine 2.4% vs control 0.6%, *p* = 0.046). Overall, these findings suggest that azvudine can be considered a safe drug that does not significantly exacerbate the occurrence of severe adverse events.

**Table 2:** Incidence of adverse events in the study population receiving conventional or Azvudine treatment.

Adverse events [N, %]	Available data		All grades			Grade ≥ 3		
	Control [1071]	Azvudine [1071]	Control	Azvudine	*p* value	Control	Azvudine	*p* value
Alanine aminotransferase (ALT)	382	549	107 (28%)	188 (34%)	0.044	17 (4.5%)	22 (4.0%)	0.7
Aspartate aminotransferase (AST)	397	563	107 (27%)	150 (27%)	>0.9	20 (5.0%)	24 (4.3%)	0.6
Alkaline phosphatase (ALP)	379	546	34 (9.0%)	45 (8.2%)	0.7	0 (0%)	0 (0%)	
Gamma‐glutamyl transpeptidase (GGT)	335	452	71 (21%)	91 (20%)	0.7	5 (1.5%)	9 (2.0%)	0.6
Uric acid (UA)	334	486	27 (8.1%)	41 (8.4%)	0.9	0 (0%)	0 (0%)	
Phosphorus (P)	210	276	49 (23%)	38 (14%)	0.006	0 (0%)	0 (0%)	
Low potassium (K)	581	636	123 (21%)	148 (23%)	0.4	33 (5.7%)	51 (8.0%)	0.11
High potassium (K)	581	636	25 (4.3%)	32 (5.0%)	0.5	0 (0%)	0 (0%)	
Cholesterol (CH)	53	104	3 (5.7%)	12 (12%)	0.2	0 (0%)	0 (0%)	
Triglyceride (TG)	43	78	8 (19%)	21 (27%)	0.3	0 (0%)	2 (2.6%)	0.5
Glucose (GLU)	96	135	15 (16%)	27 (20%)	0.4	0 (0%)	0 (0%)	
Creatinine (CREA)	384	559	57 (15%)	60 (11%)	0.06	14 (3.6%)	14 (2.5%)	0.3
Lymphocyte (Llymp)	898	887	272 (30%)	325 (37%)	0.004	155 (17%)	207 (23%)	0.001
Hlymp	898	887	18 (2.0%)	26 (2.9%)	0.2	0 (0%)	1 (0.1%)	0.5
Platelets (PLT)	415	568	87 (21%)	85 (15%)	0.014	42 (10%)	35 (6.2%)	0.023
Nrutophil (Neut)	345	466	24 (7.0%)	31 (6.7%)	0.9	2 (0.6%)	11 (2.4%)	0.046
Hemoglobin (Hb)	368	484	140 (38%)	190 (39%)	0.7	51 (14%)	64 (13%)	0.8

Abbreviations: PLT, platelets; ALT, alanine aminotransferase; AST, aspartate aminotransferase; ALP, alkaline phosphatase; CREA, creatinine; GGT, gamma‐glutamyl transpeptidase; UA, uric acid; P, phosphorus; K, potassium; CHCH, cholesterol; TG, triglyceride; Glu, glucose; Llymp, lymphocyte; Neut, neutrophil; Hb, hemoglobin.

We then further observed dynamic changes in key laboratory indicators within 15 days after azvudine administration, including key indicators of blood routine examination on immune function (**Figure** **6**A), liver function indexes (Figure 6B), kidney function indexes (Figure 6C) and coagulation function indexes (Figure 6D). Most of the indicators were basically maintained within normal range after drug treatment both in the treatment and control groups, including white blood cells, monocytes, eosinophilic granulocyte, basophilic granulocytes, ALP, TBIL, creatinine, and platelets. Regarding abnormal values on immune‐related indicators, the results indicated that during the 0–15 days period after azvudine treatment, neutrophil counts were basically above the normal range, with higher values observed in the treatment group. The lymphocyte count in the treatment group initially decreased and then increased, although remained below normal levels in both groups. Though liver and kidney‐related indicators of ALT, aspartate aminotransferase (AST), gamma‐glutamyl transpeptidase (GGT), uric acid, and urea were upper than the normal range in SARS‐CoV‐2 infected patients with concomitant liver diseases, no significant difference was observed between azvudine and control groups except for few time points.

### 2.6 Safety Evaluation in Population Coinfected with SARS‐CoV‐2 and HBV (Hepatitis B Virus)/HCV (Hepatitis C Virus)

Considering the influence of HBV/HCV coinfection on drug safety, we collected 340 eligible SARS‐CoV‐2 infected patients coinfected with HBV or HCV to evaluate the safety of azvudine. After 2:1 PSM, 110 coinfected patients and 55 matched controls were enrolled for final analysis (Table S5, Supporting Information). According to the follow‐up of key laboratory parameters for 15 days after azvudine treatment, the results demonstrated that there basically existed no significant changes between azvudine and the control group on immune‐related cells from blood routine examination (white blood cells, monocytes, basophilic granulocytes, eosinophilic granulocyte, and neutrophils) (Figure S10, Supporting Information). Though lymphocytes maintained at a relatively low level compared with control, the significance can only be detected in a few time points. No significant difference was observed in parameters related to liver function (ALT, AST, ALP, GGT) (Figure S11, Supporting Information), kidney function (creatinine, uric acid, and urea), and coagulation function (platelets) (Figure S12, Supporting Information).

## 3 Discussion

This study established a multicenter, large‐sample cohort to investigate the efficacy and safety of azvudine treatment in patients with SARS‐CoV‐2 infection complicated by liver diseases. A total of 1022 azvudine‐treated subjects and an equal number of matched conventional treatment subjects were included in the final analysis. Our findings suggested that azvudine was more effective in reducing all‐cause mortality compared to conventional treatment. Notably, azvudine exhibited a stronger effect in reducing all‐cause mortality, particularly in patients with concomitant hormone therapy, with kidney disease, and without autoimmune diseases in subgroup analysis. This may imply a significant reduction in all‐cause mortality of azvudine treatment in this subgroup, with a more significant direct association with azvudine treatment.

Regarding data analysis, compared to previous retrospective studies, this study employed three sensitivity analyses to iteratively validate the robustness of the results.^[^ ^24^, ^25^ ^]^ In all three sensitivity analyses, we performed PSM using different methods, filled up missing data using different interpolation methods, or excluded those patients may introduce bias. The results from Kaplan–Meier analysis and Cox regression analysis always remained consistent, suggesting the robustness of the effectiveness of azvudine in improving all‐cause mortality. Additionally, many laboratory test indicators may serve as potential confounding factors. Therefore, demographic characteristics, laboratory test indicators, and drug use were controlled to further ensure the accuracy and reliability of statistical results. According to subgroup analysis, azvudine showed a priority for patients receiving hormone therapy, with concomitant kidney diseases and without autoimmune diseases. As is known, steroids contribute to inhibiting inflammatory responses and reducing the risk of cytokine storms in patients infected with COVID‐19, which may provide a reasonable explanation for its effectiveness in reducing all‐cause mortality in patients receiving hormone therapy.

In a study involving 317 participants, they found that azvudine can reduce the rate of disease progression of SARS‐CoV‐2 infected patients.^[^ ^26^ ^]^ Inconsistently, in this study, azvudine treatment showed no association with reduced composite disease progression from the results of the whole population. Different sample sizes, different regions, and different population composition may provide a reasonable explanation for the difference. We thus performed further subgroups to analyze the treatment outcomes of azvudine for specific populations. Through our subgroup analysis, potential variations in the response to azvudine among specific patient cohorts were identified. Particularly noteworthy was its potentially enhanced therapeutic efficacy in patients receiving vaccination, with concomitant kidney diseases and without autoimmune diseases. These findings underscore the significance of personalized treatment approaches to maximize therapeutic outcomes and enhance patient survival rates. Despite some adverse events such as decreased lymphocyte and increased ALT levels, azvudine demonstrates an overall good safety profile, with few severe adverse events observed.

In SARS‐CoV‐2 infection, there is a positive correlation between CD4^+^ response and favorable prognosis. T cells might similarly have played a crucial role in SARS‐CoV‐2 infection.^[^ ^27^, ^28^ ^]^ The thymus, the primary immune organ responsible for generating circulating T lymphocytes, maintains overall immune function. With age, thymic atrophy significantly occurred, leading to decreased immunity and dysregulation of immune modulation. Current research on azvudine primarily focused on the thymus, indicating its importance in T cell‐mediated anti‐SARS‐CoV‐2 immunity.^[^ ^21^ ^]^

Neutrophils are the body's first line of defense against infection, swiftly responding to and eliminating pathogens. In this study, patients in the azvudine treatment group exhibited slightly higher neutrophil counts than the control group, suggesting the drug may enhance innate immune response. Moderate neutrophil response aids in rapid viral clearance, thereby alleviating symptoms and improving prognosis.^[^ ^29^ ^]^ A notable abnormality in blood during this SARS‐CoV‐2 pandemic was lymphocytopenia caused by the virus, indicating compromised immune function.^[^ ^30^, ^31^ ^]^ Furthermore, elevated cytokine levels are closely associated with lymphocytes or monocytes, being key changes in COVID‐19 and potentially triggering severe cytokine storms and death.^[^ ^32^, ^33^, ^34^, ^35^ ^]^ Impaired T‐cell regulatory function may be a significant factor leading to abnormal cytokine production.^[^ ^36^ ^]^ Thus, COVID‐19 has been shown to attack T cells, resulting in decreased lymphocyte counts, especially notable in severe cases and older patients, possibly due to compromised defensive immunity and lymphocyte or monocyte regulatory function.^[^ ^37^, ^38^ ^]^ This study found that lymphocyte counts in the treatment group exhibited a trend of initially decreasing and then increasing within 0–15 days post‐treatment. This phenomenon may reflect azvudine's early inhibitory effect on lymphocytes. Some research suggested that initial lymphocyte reduction in certain antiviral treatments might have resulted from viral suppression, lymphocyte redistribution, and direct drug effects, with treatment leading to viral load reduction and gradual immune system recovery, thus showing the process of immune reconstitution.^[^ ^39^, ^40^ ^]^ In this study, the treatment group also showed a trend of initially decreasing then increasing eosinophilic granulocyte counts, reflecting azvudine's broad impact on the immune response. Additionally, research indicated that oral azvudine could reduce inflammation, improve blood clotting function, and significantly increase eosinophil count and percentage.^[^ ^41^ ^]^ In addition, a recent study involving 290 patients confirmed, that eosinopenia was associated with worsening progression in azvudine‐treated SARS‐CoV‐2 infected patients (adjusted HR = 2.79, 95% CI: 1.04, 7.50).^[^ ^42^ ^]^ These results revealed the potential mechanisms of azvudine in immune modulation and antiviral effects, as well as its impact on patient prognosis.

Therefore, our findings on azvudine's effectiveness in reducing the mortality of SARS‐CoV‐2 infection, particularly in patients with liver issues, highlighted its therapeutic potential. Further research is crucial for elucidating the mechanisms of azvudine and customizing individualized treatments accordingly. Investigating its effects on the immune system and patient outcomes is vital for enhancing COVID‐19 management strategies.

There still exist some limitations in this study. First, because this is a retrospective cohort study based on existing real‐world data, selection bias inevitably existed. Nevertheless, we established strict inclusion criteria and collected 32 864 patients from nine hospitals in different regions, attempting to minimize the potential effect of this bias. Second, a treatment used in clinical practice can be generally affected by the clinician's treatment preferences, affordability of the patients, and availability of drugs in each hospital, which may also introduce some potential biases it is difficult to avoid afterwards. Third, all the hospitals that participated in this study were limited to Henan Province, while patient information outside the province was still insufficient. In this study, an independent cohort was used to verify the validation and generalization of our findings, but further studies in other provinces and cities are still needed to enhance the effectiveness and safety of azvudine.

## 4 Conclusion

Our multicenter, large‐sample retrospective cohort study indicated that azvudine treatment reduced the risk of all‐cause death in hospitalized SARS‐CoV‐2 infected patients with liver diseases, with few serious adverse events occurring. These findings may offer valuable real‐world evidence endorsing azvudine administration in clinical settings, reinforcing its efficacy and safety profile.

## 5 Experimental Section

### Study Design and Participants

This multicenter, retrospective cohort study, conducted across nine hospitals in Henan Province, enrolled all patients admitted with confirmed SARS‐CoV‐2 infection between December 5, 2022, and January 31, 2023. Participating hospitals included the First Affiliated Hospital of Zhengzhou University, Henan Infectious Disease Hospital, Henan Provincial Chest Hospital, Shangqiu Municipal Hospital, Luoyang Central Hospital, Nanyang Central Hospital, Guangshan County People's Hospital, the Fifth People's Hospital of Anyang, Fengqiu County People's Hospital. In addition, an independent cohort was collected from the First Affiliated Hospital of Henan University of Science & Technology. The study commenced on December 5, 2022, coinciding with China's official announcement of the relaxation of stringent SARS‐CoV‐2 infected restrictions, and concluded on January 31, 2023. Approval for the study was obtained from the Ethics Committee of the First Affiliated Hospital of Zhengzhou University (approval number: 2023‐KY‐0865‐001). The clinical trial registration number is NCT06349655 on ClinicalTrials.gov (https://clinicaltrials.gov/).

Included patients met the following criteria: 1) hospitalization with diagnosed SARS‐CoV‐2 infection and concurrent liver diseases between December 5, 2022, and January 31, 2023; 2) administration of either oral azvudine or conventional treatment alone during hospitalization. Exclusion criteria comprised: 1) age below 18 years; 2) receipt of antiviral medications other than azvudine. Diagnosis, treatment, and classification of SARS‐CoV‐2 infection were conducted in accordance with the COVID‐19 diagnosis and treatment plan (Trial Version 9 or Version 10) issued by the National Health Commission of the People's Republic of China. Eligible patients who received azvudine treatment or conventional treatment were allocated to the azvudine group or control group, respectively. Liver diseases can be categorized based on disease type into viral hepatitis, alcoholic liver disease, autoimmune liver disease, and other types. They can also be classified based on the course of the disease into acute and chronic stages, and further refined based on liver function, complications, and laboratory test results. This study encompassed 17 types of liver diseases including hepatic cyst, polycystic liver, liver nodule, liver abscess, hepatic hemangioma, hepatitis, liver cirrhosis, fatty liver, hepatic calculus, liver tumor, liver failure, hepatic encephalopathy, hepatic insufficiency, liver injury, liver congestion, hepatic veno‐occlusive disease (HVOD), and hepatic myelopathy.

### Data Source

The entirety of the study's data emanated from electronic medical records extracted from the ten hospitals situated within Henan Province. These records encompassed: 1) demographic particulars, gender, age, and body mass index (BMI); 2) medical history; 3) clinical documentation, inclusive of admission particulars, diagnoses, prescribed treatments, laboratory analyses, imaging findings, intensive care unit (ICU) admissions, and discharge or mortality dates.

### Treatment Exposure

In this study, patients hospitalized for SARS‐CoV‐2 infection combined with liver diseases took 5 mg of azvudine orally once a day. The control subjects were selected from hospitalized patients with SARS‐CoV‐2 combined with liver diseases, who only received conventional treatment during the study period and did not receive any antiviral drugs.

### Outcomes

The main outcome was all‐cause death, while the secondary outcome was given to composite disease progression. Composite disease progression was determined through electronic health records. Composite disease progression encompassed a range of factors indicating increased severity, such as death, commencement of high‐flow nasal cannula (HFNC) oxygen therapy, introduction of invasive or non‐invasive mechanical ventilation, and admission to the ICU.

### Safety

Safety outcomes were assessed by examining adverse events of all severities and those graded as ≥ 3. Adverse events primarily consisted of abnormal laboratory findings, as defined by the Common Terminology Criteria for Adverse Events, Version 5.0 (CTCAE 5.0). Data were collected from the initiation of azvudine treatment until 5 half‐lives post the final dose. In cases of multiple abnormalities, the most serious result was chosen for analysis.

### Baseline Covariates

Baseline characteristics encompassed various factors: age, gender, BMI, and the severity of SARS‐CoV‐2 infection at admission. Severity gradations were as follows: mild cases exhibited typical respiratory symptoms, moderate cases featured persistent high fever (>3 days) with a respiratory rate <30 times per minute or oxygen saturation >93%, while severe cases manifested with a respiratory rate ≥30 times per minute, or oxygen saturation ≤93% at rest, or PaO2/FiO2 ≤300 mmHg, or lung infiltrates >50%. Additionally, vaccination status, concomitant hormone or antibiotics treatments at diagnosis, and comorbidities such as diabetes, hypertension, cardio‐cerebral diseases, chronic kidney diseases, cancer, chronic respiratory diseases, and autoimmune diseases were considered. Moreover, pertinent laboratory parameters at diagnosis were recorded, including neutrophil (Neut), lymphocyte (Lymph), procalcitonin (PCT), activated partial thromboplastin time (APTT), prothrombin time (PT), cholesterol (CH), C‐reactive protein (CRP), glucose (Glu), high‐density lipoprotein (HDL), ALT, GGT, AST, ALP, creatinine (CREA), albumin (ALB), triglyceride (TG), low‐density lipoprotein (LDL), glomerular filtration rate (e‐GFR), and total bilirubin (TBIL).

### Sensitivity Analysis

In sensitivity analysis, Probit regression was employed to align propensity scores, utilized mean imputation to address missing data, or excluded populations who were discharged or died on the medication initiation day for a refined validation of findings. Subsequently, Kaplan–Meier analysis and Cox regression modeling were performed to assess the robustness of the research outcomes.

### Statistical Analysis

To mitigate confounding variables and baseline covariate discrepancies, a 1:1 propensity score matching (PSM) technique was utilized to identify matched subjects in both the azvudine and control cohorts employing a probabilistic approach. Multiple imputation was employed to address missing data in baseline characteristics. Following propensity score matching, baseline covariates between the azvudine and control groups were aligned, with a balance confirmed for *p*>0.05. The overall cumulative hazards were estimated using the Kaplan–Meier method, with statistical comparisons facilitated by the log‐rank test between the azvudine and control cohorts. To investigate variables linked to primary outcomes, a Cox proportional hazards regression model (Cox regression) was employed to ascertain HR alongside 95% CI. A HR greater than 1 denoted an elevated risk, while a HR less than 1 implied a reduced risk. The proportional hazards assumption was assessed using Schoenfeld residuals, and multicollinearity was evaluated using the variance inflation factor (VIF), where a VIF value >5 indicated multicollinearity. The occurrence of adverse events was presented as proportions or interquartile range (IQR), with statistical differences determined via Pearson's Chi‐squared test or Wilcoxon rank sum test, respectively.

Subgroup analyses were performed across each level of the baseline covariates to validate the estimates' robustness. All statistical analyses were conducted using R software (version 4.3.0, R Foundation for Statistical Computing). Statistical significance was defined as a two‐sided *p*<0.05.

### Ethics Approval

This study gained approval from the Institutional Review Board of The First Affiliated Hospital of Zhengzhou University (2023‐KY‐0865‐001).

## Conflict of Interest

The authors declare no conflict of interest.

## Author Contributions

J.S., M.Y., G.S., L.W, and X.H. contributed equally to this work. R.Z.G. and Y.Z.J. performed conceptualization, data curation, investigation, methodology, project administration, resources, and writing review and editing. S.J.Y., Y.M.Z., S.G.Y., W.L., C.G.Y., Z.Y.J., and H.X.B. performed investigation and writing‐original draft. Q.G.W., Y.Y.Q., H.X.J., L.S.L., L.H., Z.S.X., L.G.M., Z.D.H., and L.G.T. performed formal analysis, investigation, and resources. Y.M.Z. and C.M. performed formal analysis and software. All authors had full access to all the data in the study and agreed to submit the manuscript for publication.

## Supporting information



Supporting Information

## Data Availability

The data that support the findings of this study are available from the corresponding author upon reasonable request.
