# Supplementary material for: The Antiviral Efficacy and Safety of Azvudine in Hospitalized SARS‐CoV‐2 Infected Patients with Liver Diseases Based on a Multicenter, Retrospective Cohort Study
Source: Adv Sci (Weinh). 2025 Feb 22;12(15):2405679. doi: 10.1002/advs.202405679 (PMC12005779; doi:10.1002/advs.202405679)
Supplement: Supplementary file 1 — Supporting Information [file ADVS-12-2405679-s001.docx]

**Supplementary Materials**

**Title:** The antiviral efficacy and safety of azvudine in hospitalized SARS-CoV-2 infected patients with liver diseases based on a multicenter, retrospective cohort study

**Authors**

Junyi Sun ^1†^, Mengzhao Yang ^1†^, Guanyue Su ^1†^, Ling Wang ^2†^, Xiaobo Hu ^1†^, Yongjian Zhou ^1^, Guangying Cui ^1^, Guowu Qian ^3^, Yiqiang Yuan ^2^, Xinjun Hu ^4^, Silin Li ^5^, Hong Luo ^6^, Shixi Zhang ^7^, Guangming Li ^8^, Donghua Zhang ^9^, Guotao Li ^10^, Ming Cheng ^11^, Zujiang Yu^1^*, Zhigang Ren^1^*

**Affiliations**

^1^ Department of Infectious Diseases, State Key Laboratory of Antiviral Drugs, Pingyuan Laboratory, the First Affiliated Hospital of Zhengzhou University, Zhengzhou 450052, China;

^2^ Department of Cardiovascular Medicine, Henan Provincial Chest Hospital Affiliated to Zhengzhou University, Zhengzhou 450008, China;

^3^ Department of Gastrointestinal Surgery, Nanyang Central Hospital, Nanyang 473009, China;

^4^ Department of Infectious Diseases, The First Affiliated Hospital, College of Clinical Medicine, Henan University of Science and Technology, Luoyang, 471003, China;

^5^ Department of Respiratory and Critical Care Medicine, Fengqiu County People's Hospital, Xinxiang 453300, China;

^6^ Guangshan County People’s Hospital, Guangshan County, Xinyang 465450, China;

^7^ Department of Infectious Diseases, Shangqiu Municipal Hospital, Shangqiu 476000, China;

^8^ Department of Liver Disease, the Affiliated Infectious Disease Hospital of Zhengzhou University, Zhengzhou 450052, China;

^9^ Department of Infectious Diseases, Anyang City Fifth People’s Hospital, Anyang 455000, China;

^10^ Department of Infectious Diseases, Luoyang Central Hospital Affiliated to Zhengzhou University, Luoyang 471000, China;

^11^ Department of Medical Information, the First Affiliated Hospital of Zhengzhou University, Zhengzhou 450052, China;

^†^ These authors contributed equally to this work.

^*^**Address correspondence to:**

Zhigang Ren, Ph.D, M.D., Department of Infectious Diseases, State Key Laboratory of Antiviral Drugs, Pingyuan Laboratory, the First Affiliated Hospital of Zhengzhou University, #1 Jianshe East Road, Zhengzhou 450052, China. E-mail: [fccrenzg@zzu.edu.cn](mailto:fccrenzg@zzu.edu.cn)

Zujiang Yu, Prof., M.D., Department of Infectious Diseases, State Key Laboratory of Antiviral Drugs, Pingyuan Laboratory, the First Affiliated Hospital of Zhengzhou University, #1 Jianshe East Road, Zhengzhou 450052, China. E-mail: [johnyuem@zzu.edu.cn](mailto:johnyuem@zzu.edu.cn)

# Supplementary figures


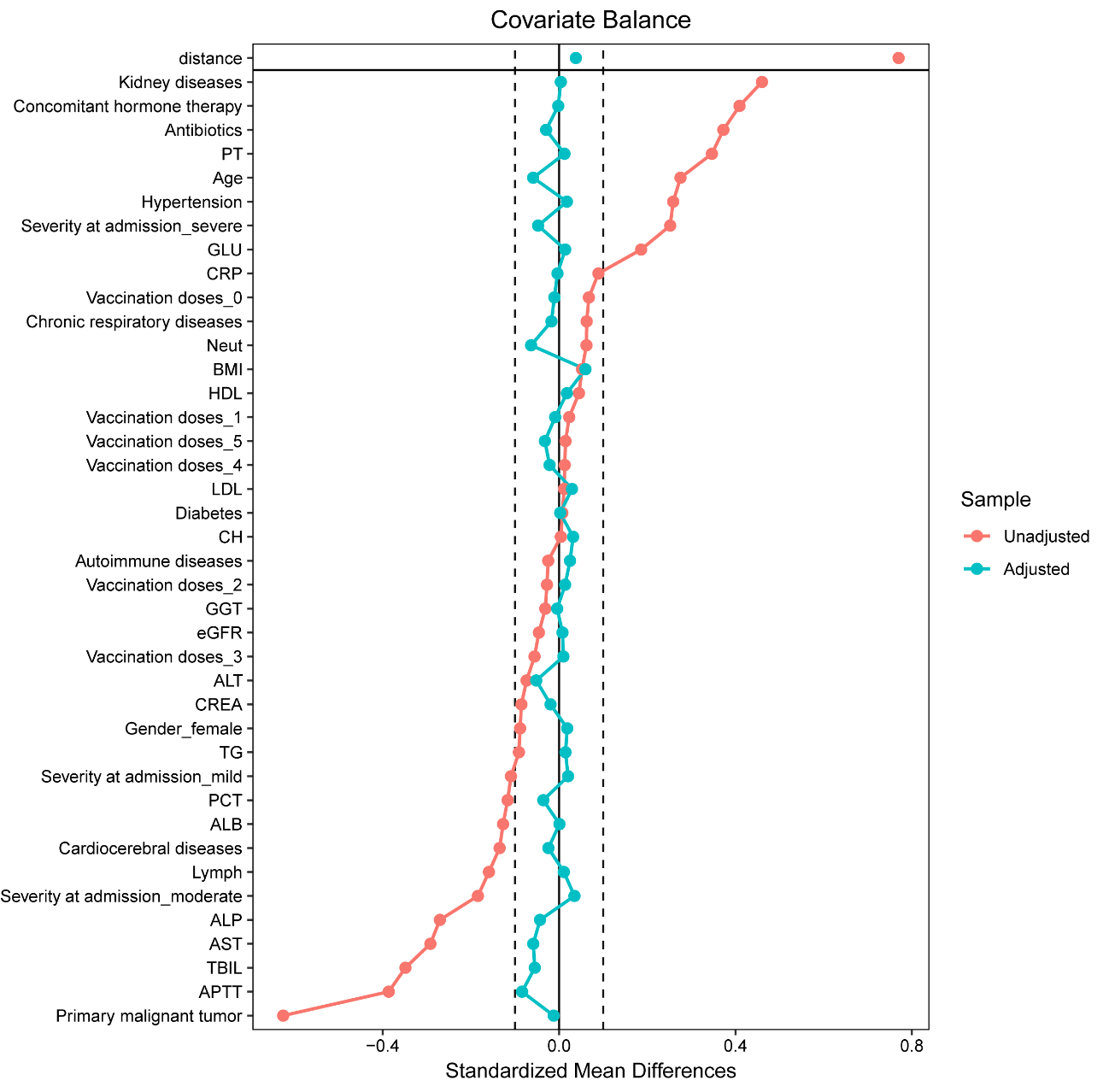
Figure S1 Baseline characteristics before and after propensity score matching


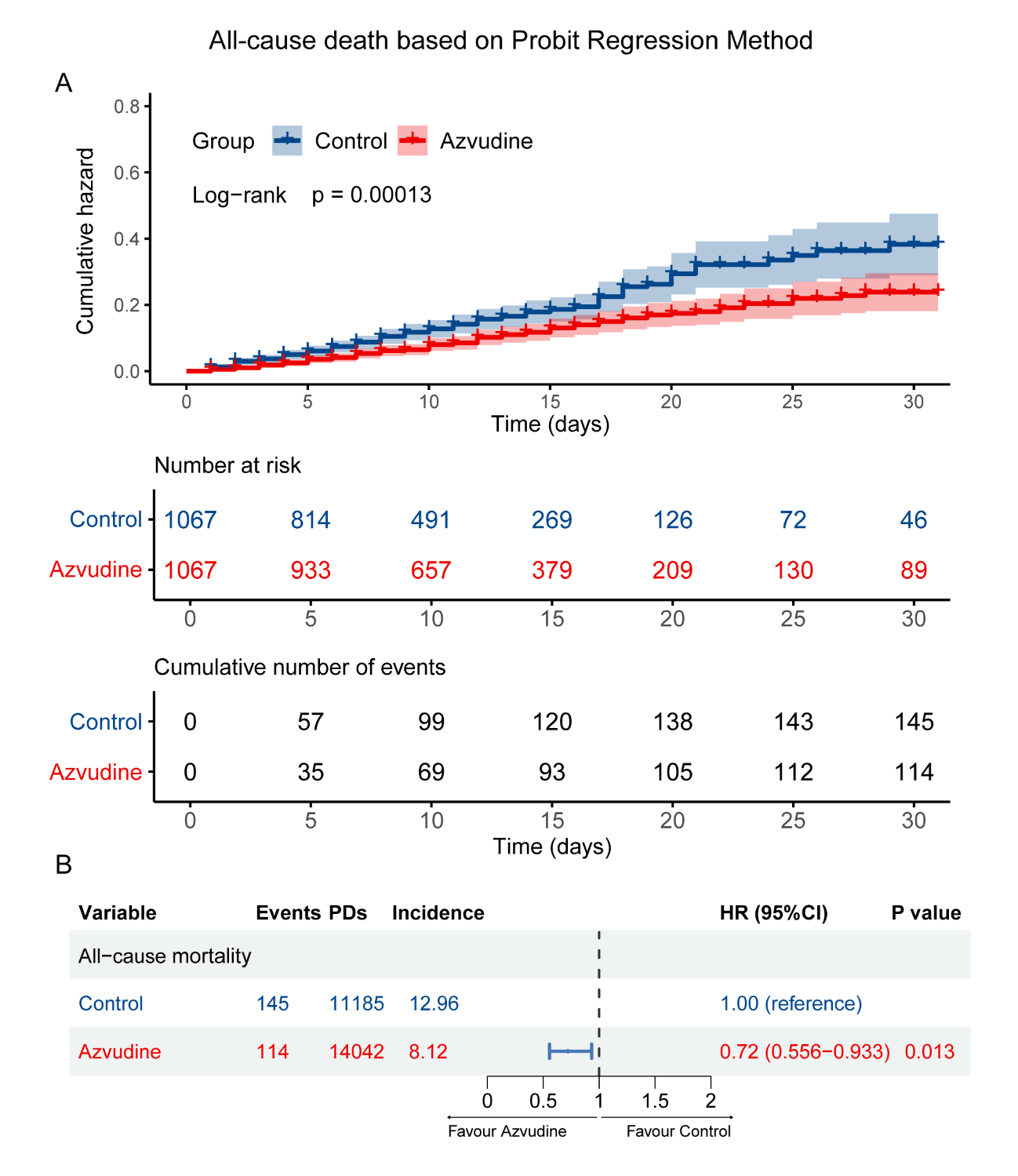


Figure S2 Effectiveness of azvudine treatment in reducing all-cause death where propensity score matching was performed using Probit regression method. (A) Kaplan-Meier curves; (B) Multivariate Cox proportional hazards regression analysis.


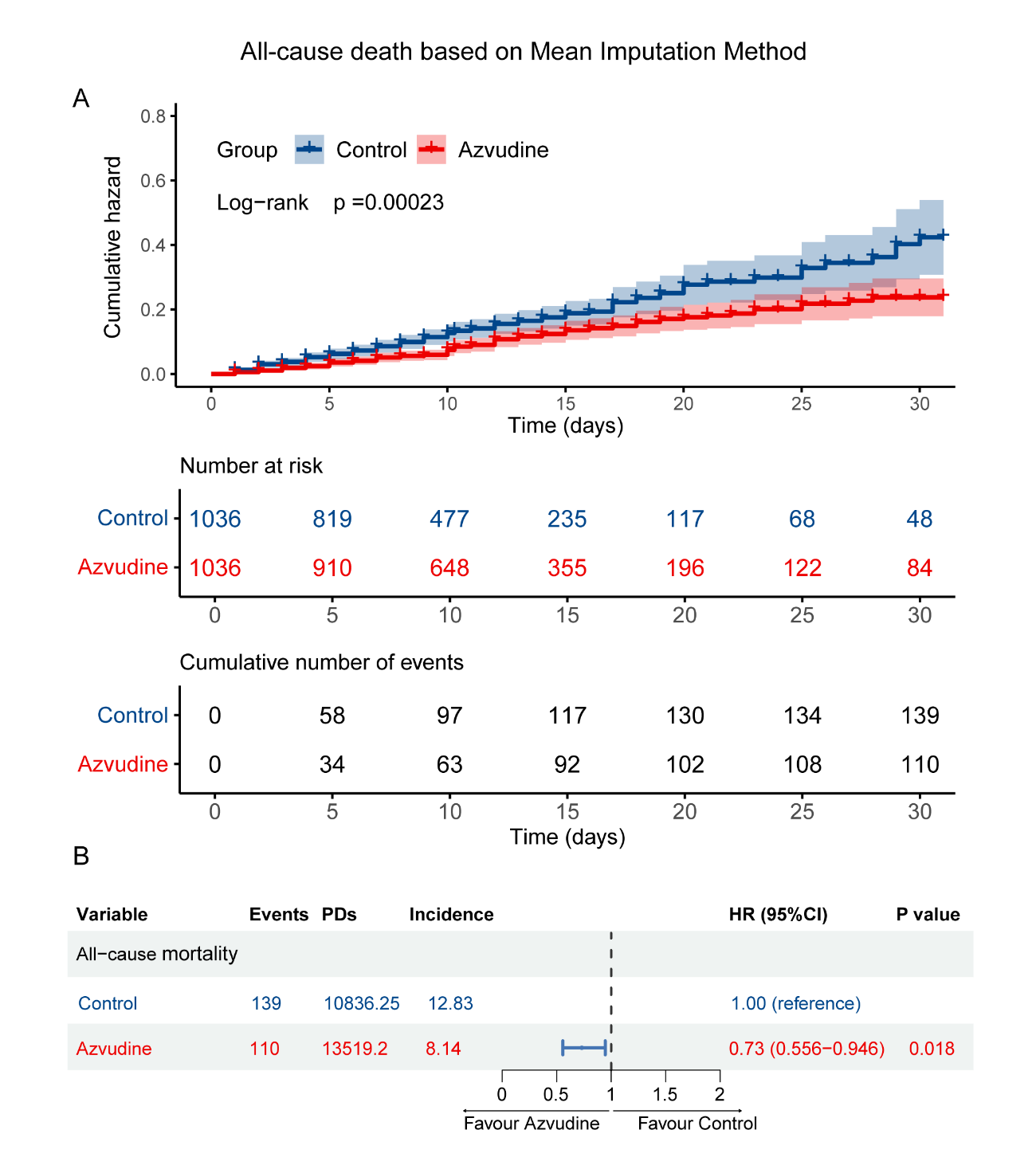


Figure S3 Effectiveness of azvudine treatment in reducing all-cause death where the missing data was filled up using mean imputation method. (A) Kaplan-Meier curves; (B) Multivariate Cox proportional hazards regression analysis.


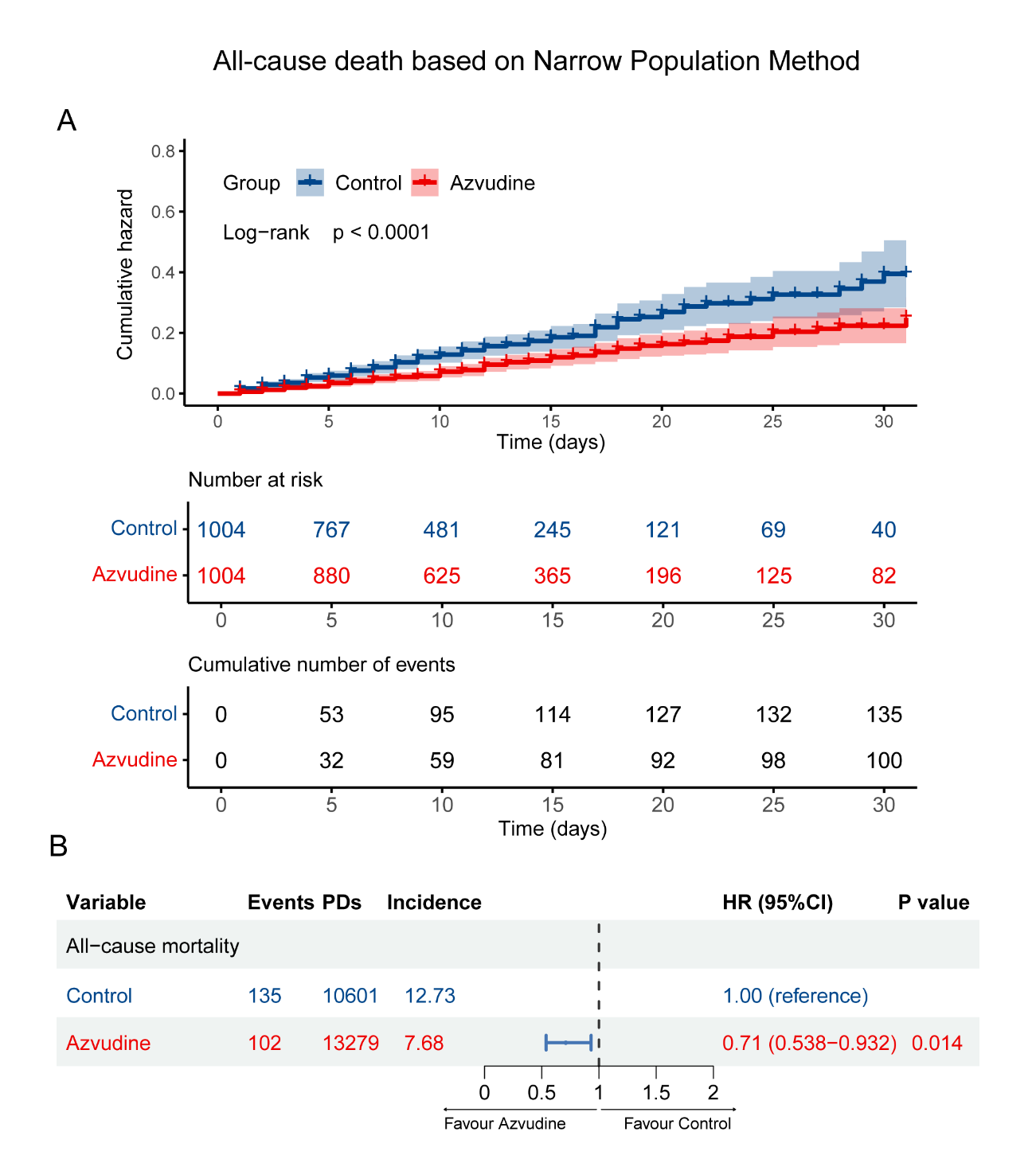


Figure S4 Effectiveness of azvudine treatment in reducing all-cause death where patients who discharged within one day after receiving antiviral treatment were excluded. (A) Kaplan-Meier curves; (B) Multivariate Cox proportional hazards regression analysis.


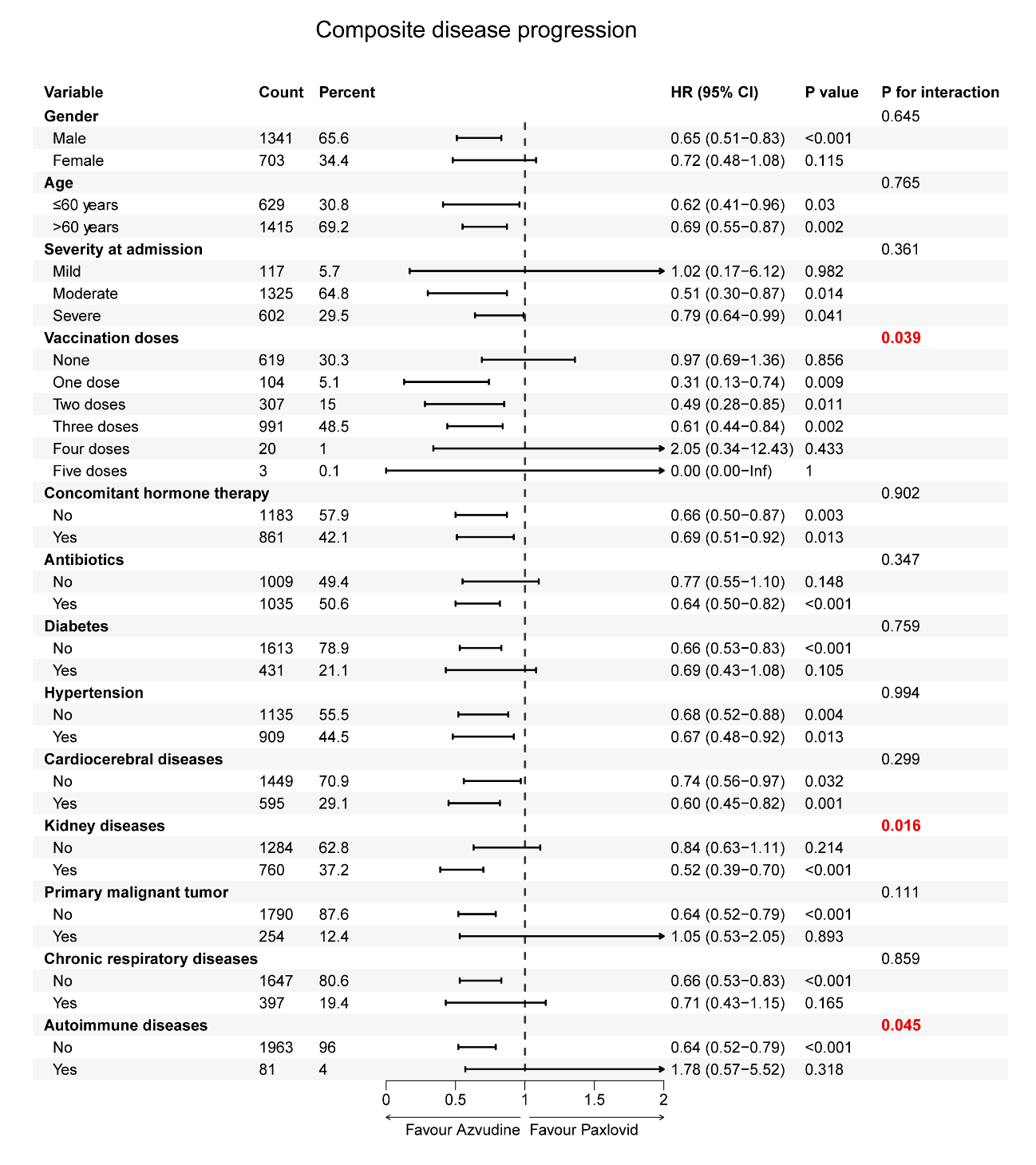


Figure S5 Effectiveness of azvudine in reducing the risk of composite disease progression by subgroups of selected baseline characteristics. HR: Hazard Ratio; 95%CI: 95% confidence interval.


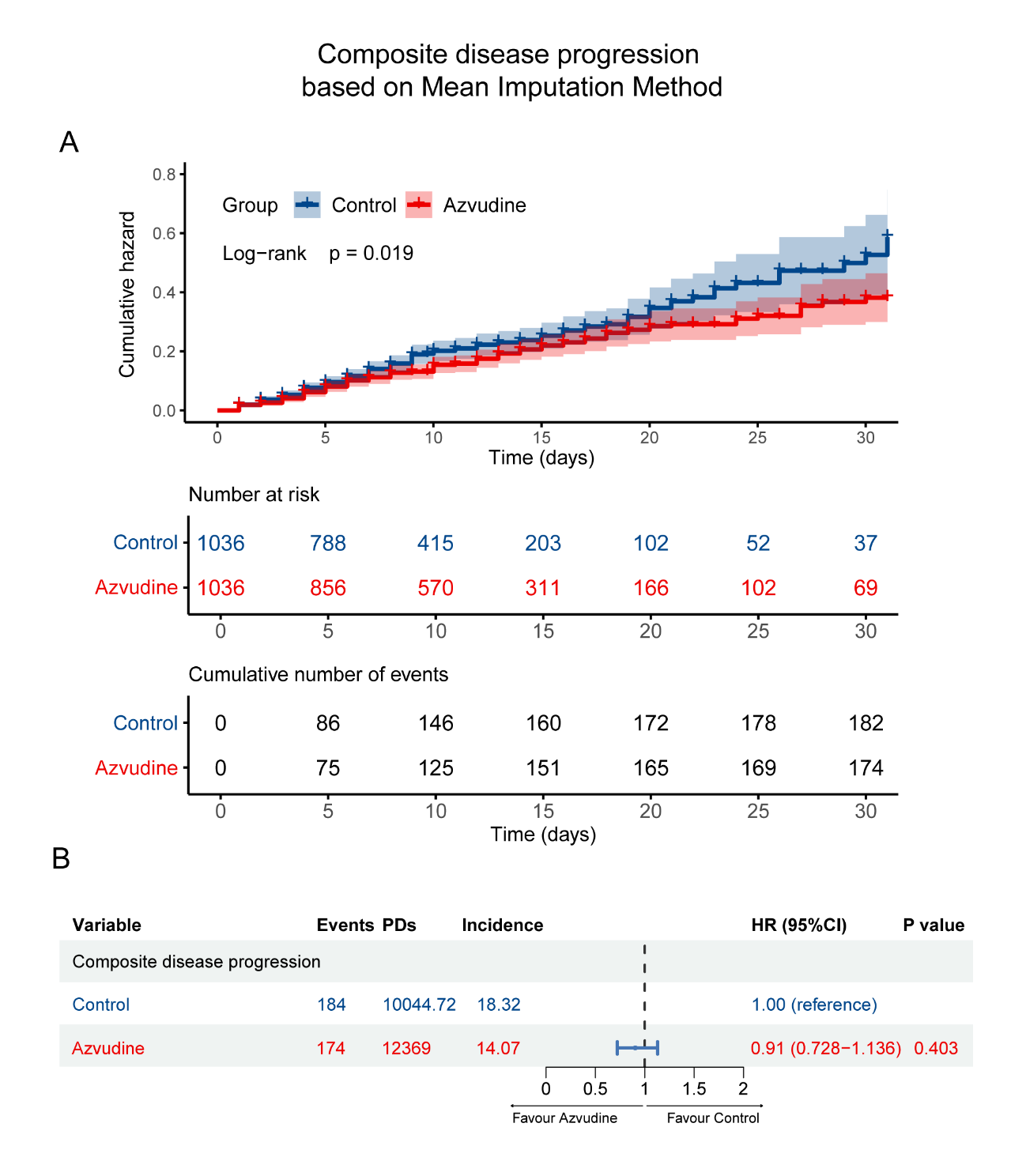


Figure S6 Effectiveness of azvudine treatment in reducing composite disease progression where the missing data was filled up using mean imputation method. (A) Kaplan–Meier curves; (B) Multivariate Cox proportional hazards regression analysis.


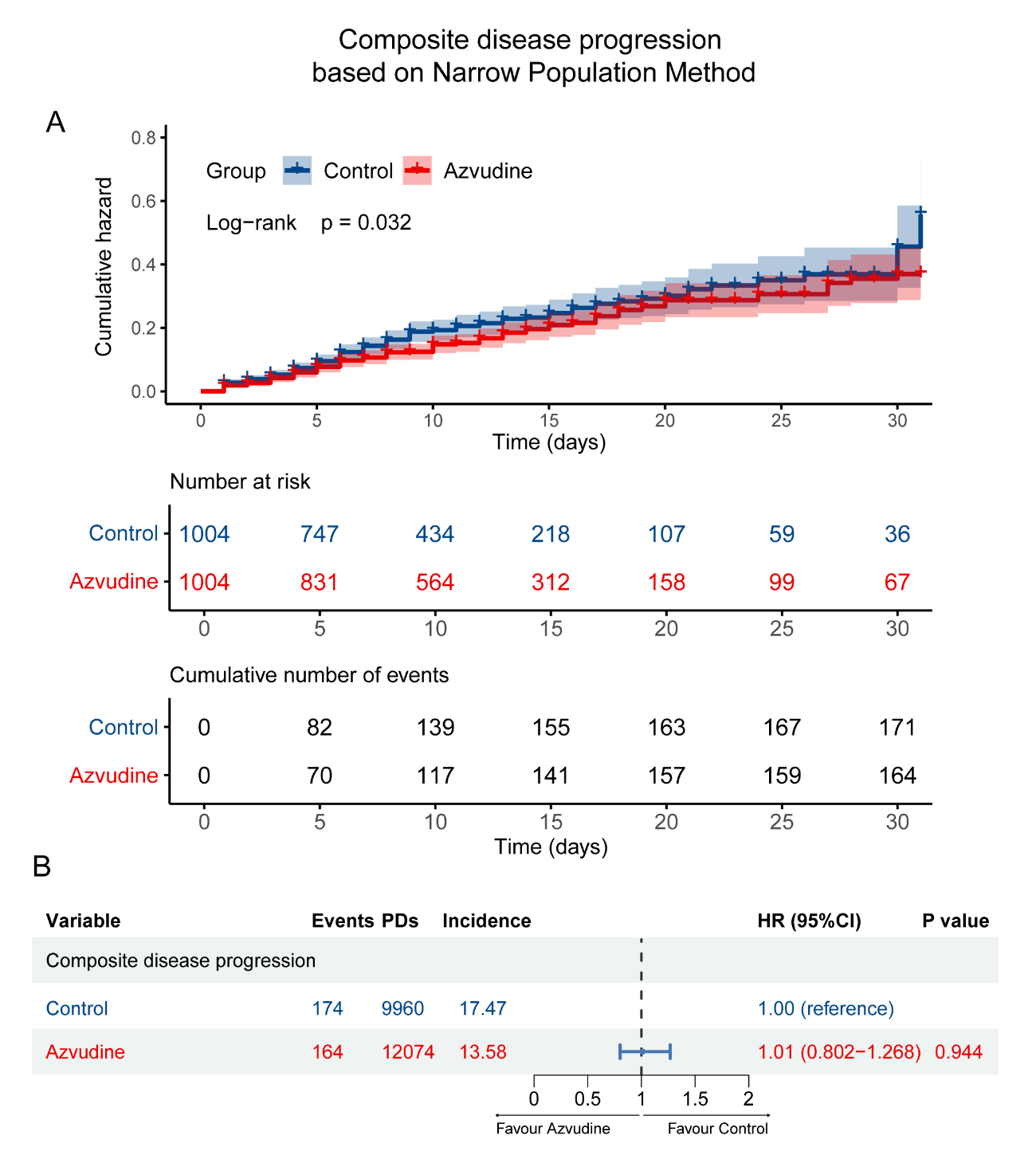


Figure S7 Effectiveness of azvudine treatment in reducing composite disease progression where patients who discharged within one day after receiving antiviral treatment were excluded. (A) Kaplan-Meier curves; (B) Multivariate Cox proportional hazards regression analysis.


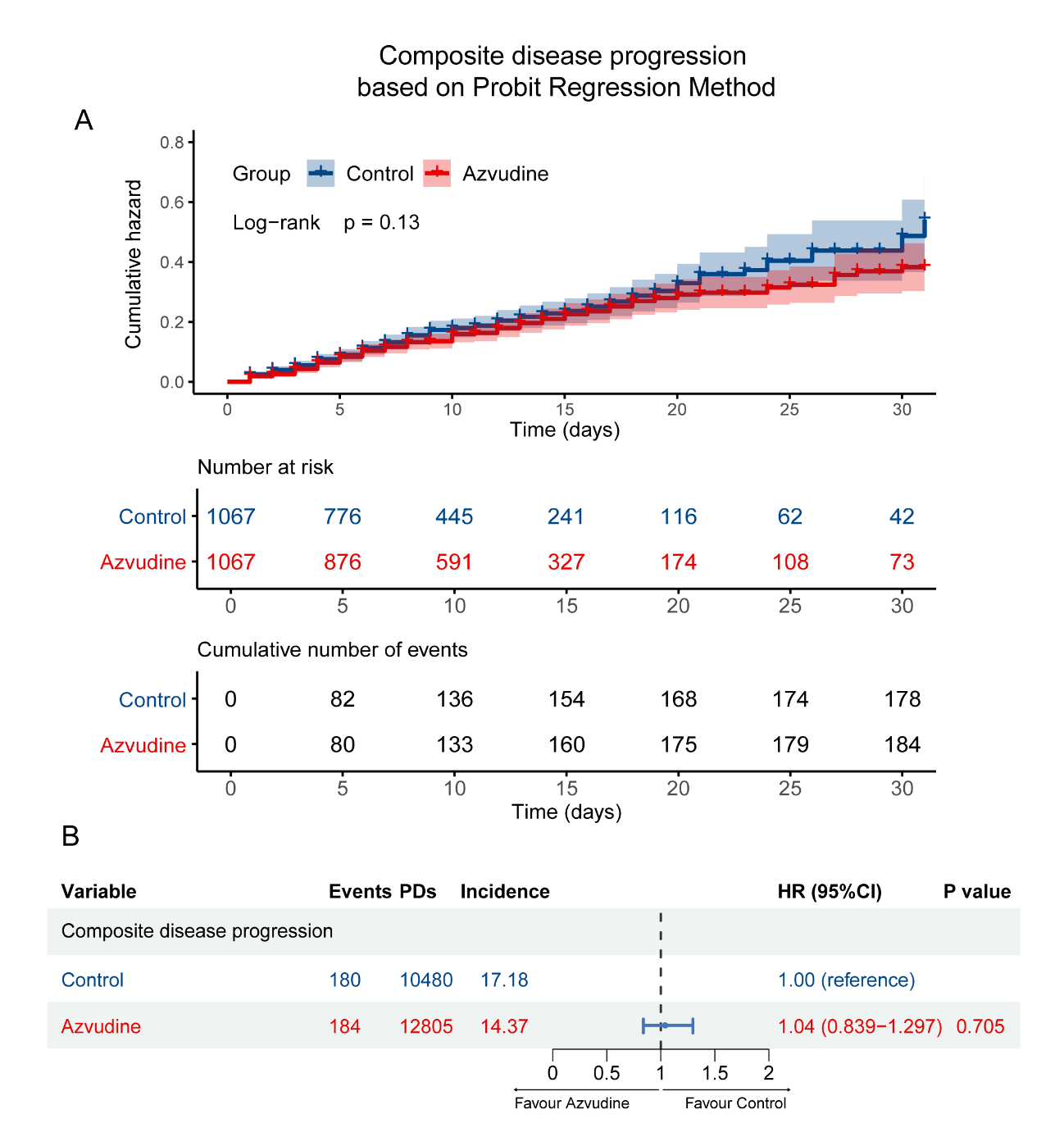


Figure S8 Effectiveness of azvudine treatment in reducing composite disease progression where propensity score matching was performed using Probit regression method. (A) Kaplan-Meier curves; (B) Multivariate Cox proportional hazards regression analysis.


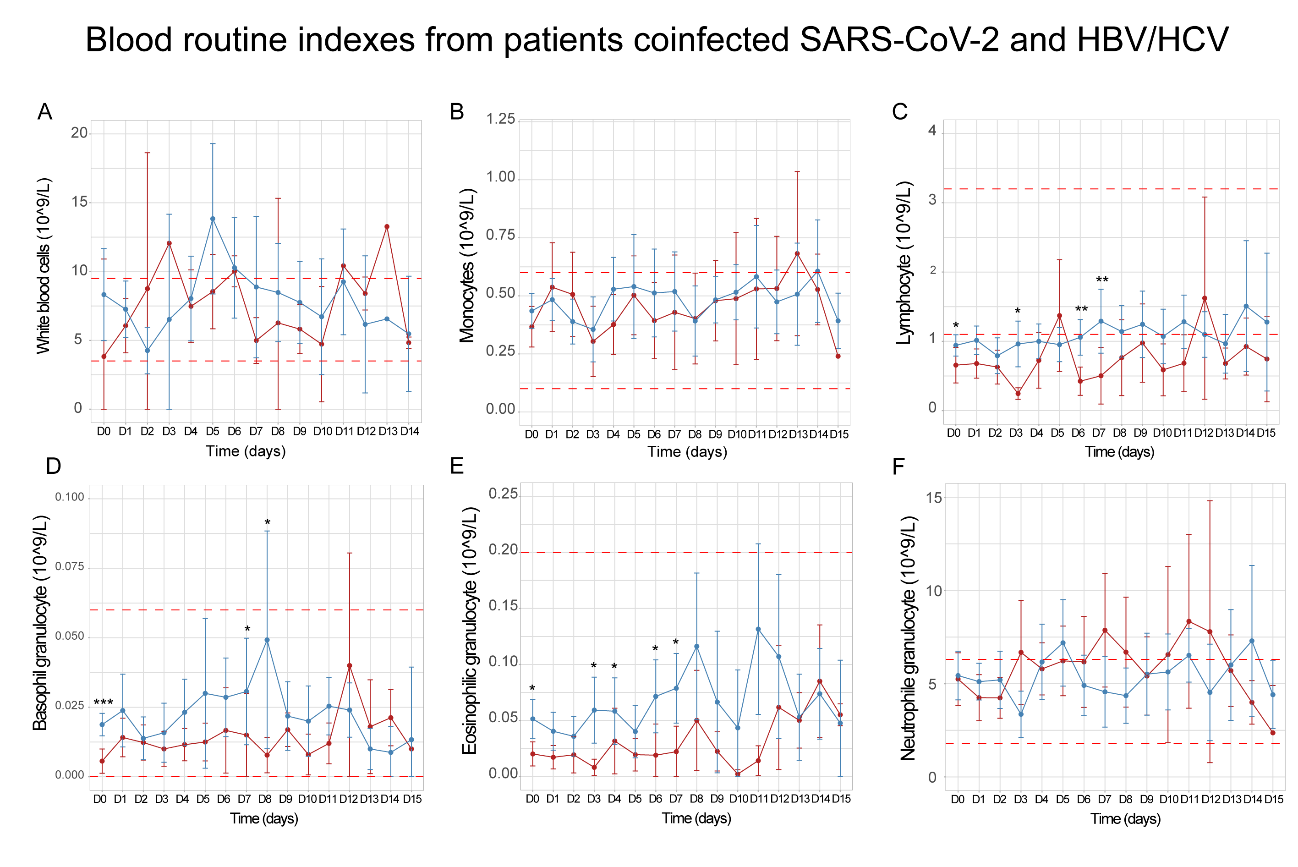


Figure S9 Dynamic changes of key blood routine indexes within 15 days after taking azvudine in SARS-CoV-2 infected patients coinfected with HBV/HCV.


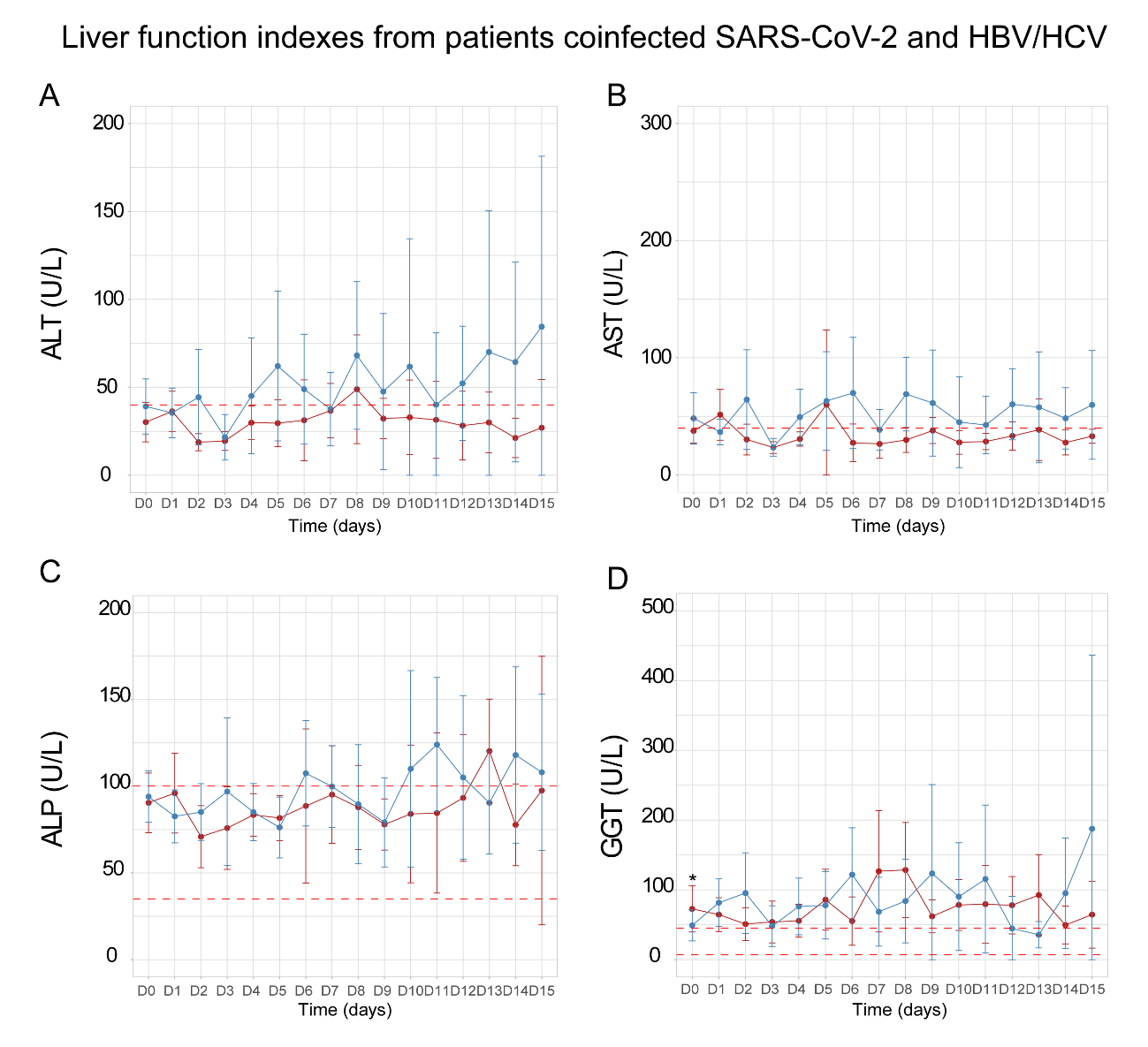


Figure S10 Dynamic changes of key indexes on liver function within 15 days after taking azvudine in SARS-CoV-2 infected patients coinfected with HBV/HCV.


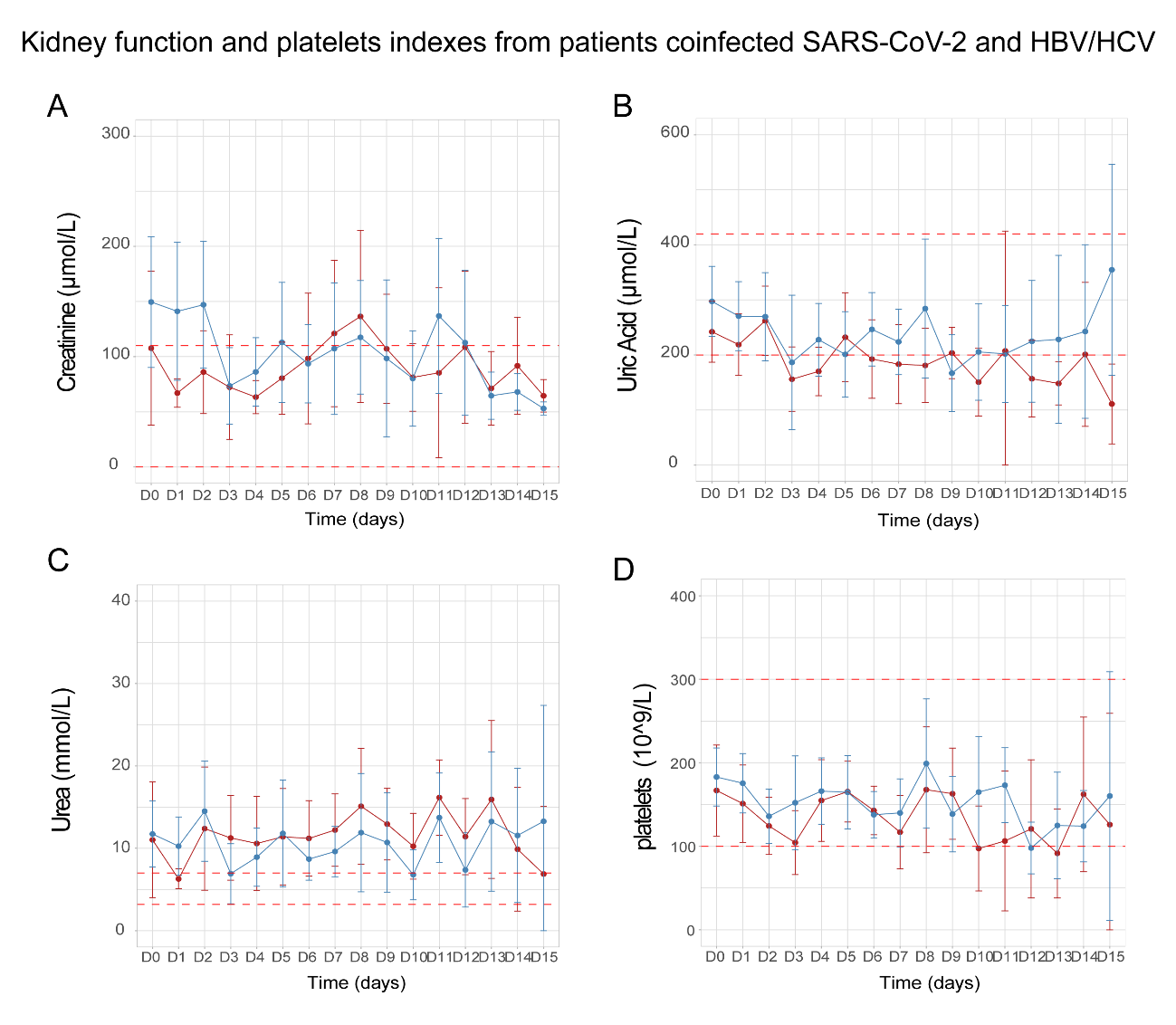


Figure S11 Dynamic changes of key indexes on kidney function and platelets within 15 days after taking azvudine in SARS-CoV-2 infected patients coinfected with HBV/HCV.

# Supplementary tables

Table S1 Baseline characteristics of the population in the sensitivity analysis where propensity score matching was performed using Probit regression method

| Baseline characteristics | Before matching | | |  | After propensity score matching (1:1) | | |
| --- | --- | --- | --- | --- | --- | --- | --- |
|  | Control  （n=4201） | Azvudine  (n=1084) | *P* value |  | Control  （n=1067） | Azvudine  (n=1067) | *P* value |
| Sociodemographic information |  |  |  |  |  |  |  |
| Age, years (mean ± SD) | 62.57 (14.51) | 66.51 (14.34) | <0.001 |  | 66.31 (14.85) | 66.41 (14.34) | 0.873 |
| Gender, n(%) |  |  | 0.012 |  |  |  | 0.717 |
| Male | 2567 (61.1) | 708 (65.3) |  |  | 686 (64.3) | 695 (65.1) |  |
| Female | 1634 (38.9) | 376 (34.7) |  |  | 381 (35.7) | 372 (34.9) |  |
| BMI, kg/m^2^ (mean ± SD) | 24.15 (3.97) | 24.36 (4.03) | 0.121 |  | 24.26 (3.90) | 24.36 (4.02) | 0.551 |
| Severity at admission, n(%) |  |  | <0.001 |  |  |  | 0.732 |
| Mild | 370 ( 8.8) | 67 ( 6.2) |  |  | 74 ( 6.9) | 65 ( 6.1) |  |
| Moderate | 3101 (73.8) | 705 (65.0) |  |  | 686 (64.3) | 693 (64.9) |  |
| Severe | 730 (17.4) | 312 (28.8) |  |  | 307 (28.8) | 309 (29.0) |  |
| Vaccination doses (%) |  |  | 0.331 |  |  |  | 0.373 |
| Unvaccinated | 1137 (27.1) | 327 (30.2) |  |  | 362 (33.9) | 321 (30.1) |  |
| One dose | 192 ( 4.6) | 55 ( 5.1) |  |  | 55 ( 5.2) | 55 ( 5.2) |  |
| Two doses | 669 (15.9) | 162 (14.9) |  |  | 152 (14.2) | 161 (15.1) |  |
| Three doses | 2171 (51.7) | 530 (48.9) |  |  | 493 (46.2) | 520 (48.7) |  |
| Four doses | 30 ( 0.7) | 9 ( 0.8) |  |  | 4 ( 0.4) | 9 ( 0.8) |  |
| Five doses | 2 ( 0.0) | 1 ( 0.1) |  |  | 1 ( 0.1) | 1 ( 0.1) |  |
| Concomitant systemic steroid, n(%) |  |  | <0.001 |  |  |  | 0.161 |
| No | 3165 (75.3) | 596 (55.0) |  |  | 628 (58.9) | 595 (55.8) |  |
| Yes | 1036 (24.7) | 488 (45.0) |  |  | 439 (41.1) | 472 (44.2) |  |
| Antibiotics, n(%) |  |  | <0.001 |  |  |  | 0.931 |
| No | 2801 (66.7) | 521 (48.1) |  |  | 520 (48.7) | 517 (48.5) |  |
| Yes | 1400 (33.3) | 563 (51.9) |  |  | 547 (51.3) | 550 (51.5) |  |
| Comorbidities, n(%) |  |  |  |  |  |  |  |
| Diabetes |  |  | 0.861 |  |  |  | 1 |
| No | 3357 (79.9) | 863 (79.6) |  |  | 847 (79.4) | 847 (79.4) |  |
| Yes | 844 (20.1) | 221 (20.4) |  |  | 220 (20.6) | 220 (20.6) |  |
| Hypertension |  |  | <0.001 |  |  |  | 0.632 |
| No | 2809 (66.9) | 585 (54.0) |  |  | 593 (55.6) | 581 (54.5) |  |
| Yes | 1392 (33.1) | 499 (46.0) |  |  | 474 (44.4) | 486 (45.5) |  |
| Cardio-cerebral diseases |  |  | <0.001 |  |  |  | 0.095 |
| No | 2818 (67.1) | 792 (73.1) |  |  | 739 (69.3) | 775 (72.6) |  |
| Yes | 1383 (32.9) | 292 (26.9) |  |  | 328 (30.7) | 292 (27.4) |  |
| Kidney diseases |  |  | <0.001 |  |  |  | 0.79 |
| No | 3438 (81.8) | 642 (59.2) |  |  | 649 (60.8) | 642 (60.2) |  |
| Yes | 763 (18.2) | 442 (40.8) |  |  | 418 (39.2) | 425 (39.8) |  |
| Primary malignant tumor |  |  | <0.001 |  |  |  | 0.892 |
| No | 2877 (68.5) | 959 (88.5) |  |  | 945 (88.6) | 942 (88.3) |  |
| Yes | 1324 (31.5) | 125 (11.5) |  |  | 122 (11.4) | 125 (11.7) |  |
| Chronic respiratory diseases, n(%) |  |  | 0.062 |  |  |  | 0.619 |
| No | 3491 (83.1) | 874 (80.6) |  |  | 870 (81.5) | 860 (80.6) |  |
| Yes | 710 (16.9) | 210 (19.4) |  |  | 197 (18.5) | 207 (19.4) |  |
| Autoimmune diseases, n(%) |  |  | 0.541 |  |  |  | 0.747 |
| No | 4010 (95.5) | 1040 (95.9) |  |  | 1020 (95.6) | 1024 (96.0) |  |
| Yes | 191 ( 4.5) | 44 ( 4.1) |  |  | 47 ( 4.4) | 43 ( 4.0) |  |
| Laboratory parameters, (mean ± SD) |  |  |  |  |  |  |  |
| Neutrophil, ×10^9^/L | 5.79 (4.68) | 6.04 (4.06) | 0.104 |  | 6.17 (4.48) | 6.07 (4.08) | 0.59 |
| Lymphocyte, ×10^9^/L | 1.25 (2.36) | 1.05 (1.24) | 0.008 |  | 1.04 (0.70) | 1.06 (1.24) | 0.713 |
| Glucose, mmol/L | 7.16 (3.85) | 7.90 (3.99) | <0.001 |  | 7.83 (4.38) | 7.89 (4.00) | 0.714 |
| High-density lipoprotein, mmol/L | 1.07 (1.79) | 1.20 (2.71) | 0.074 |  | 1.28 (3.24) | 1.16 (2.44) | 0.324 |
| Low-density lipoprotein, mmol/L | 2.35 (1.72) | 2.37 (2.16) | 0.688 |  | 2.40 (2.02) | 2.38 (2.18) | 0.827 |
| Alanine aminotransferase, IU/L | 64.41 (222.36) | 56.65 (105.38) | 0.264 |  | 59.34 (207.21) | 56.92 (106.16) | 0.734 |
| Aspartate aminotransferase, IU/L | 82.91 (371.25) | 56.03 (92.12) | 0.018 |  | 58.90 (115.17) | 56.33 (92.80) | 0.571 |
| Creatinine, µmol/L | 116.33 (324.81) | 100.48 (185.61) | 0.123 |  | 107.29 (162.40) | 100.89 (187.03) | 0.398 |
| glomerular filtration rate, ml/min | 118.04 (165.32) | 111.19 (149.25) | 0.216 |  | 108.16 (137.89) | 111.52 (150.37) | 0.591 |
| C–reactive protein, mg/L | 50.61 (66.68) | 56.82 (69.58) | 0.007 |  | 58.22 (72.17) | 56.96 (69.93) | 0.682 |
| Procalcitonin, ng/ml | 2.13 (10.80) | 1.30 (7.15) | 0.016 |  | 1.43 (6.02) | 1.31 (7.21) | 0.69 |
| Prothrombin time, s | 15.10 (8.67) | 18.60 (10.10) | <0.001 |  | 18.19 (10.37) | 18.43 (10.04) | 0.599 |
| Activated partial thromboplastin time, s | 29.46 (9.70) | 25.12 (11.24) | <0.001 |  | 26.22 (9.42) | 25.31 (11.21) | 0.043 |
| Cholesterol, mmol/L | 4.22 (5.98) | 4.24 (6.03) | 0.907 |  | 4.40 (8.20) | 4.24 (6.08) | 0.609 |
| Triglyceride, mmol/L | 1.76 (4.09) | 1.54 (2.38) | 0.094 |  | 1.57 (2.29) | 1.54 (2.38) | 0.755 |
| Alkaline phosphatase, IU/L | 116.80 (130.29) | 96.01 (76.91) | <0.001 |  | 103.39 (90.35) | 96.40 (77.43) | 0.055 |
| Gamma-glutamyl transpeptidase, IU/L | 91.62 (154.68) | 85.19 (205.50) | 0.256 |  | 87.01 (142.65) | 85.53 (207.04) | 0.848 |
| Albumin, g/L | 36.16 (11.21) | 34.51 (12.96) | <0.001 |  | 34.62 (10.90) | 34.52 (13.05) | 0.852 |
| Total bilirubin, umol/L | 24.49 (55.32) | 15.69 (25.23) | <0.001 |  | 17.58 (28.91) | 15.73 (25.42) | 0.117 |

**Abbreviations:** BMI, Body mass index.

Table S2 Baseline characteristics of the population in the sensitivity analysis where the missing data was filled up using mean imputation method

| Baseline characteristics | Before matching | | |  | After propensity score matching (1:1) | | |
| --- | --- | --- | --- | --- | --- | --- | --- |
|  | Control  （n=4201） | Azvudine  (n=1084) | *P* value |  | Control  （n=1036） | Azvudine  (n=1036) | *P* value |
| Sociodemographic information |  |  |  |  |  |  |  |
| Age, years (mean ± SD) | 62.56 (14.51) | 66.51 (14.34) | <0.001 |  | 65.70 (15.10) | 66.25 (14.28) | 0.39 |
| Gender, n(%) |  |  | 0.012 |  |  |  | 0.578 |
| Male | 2566 (61.1) | 708 (65.3) |  |  | 691 (66.7) | 678 (65.4) |  |
| Female | 1635 (38.9) | 376 (34.7) |  |  | 345 (33.3) | 358 (34.6) |  |
| BMI, kg/m^2^ (mean ± SD) | 24.21 (2.81) | 24.42 (2.51) | 0.021 |  | 24.42 (2.85) | 24.43 (2.54) | 0.876 |
| Severity at admission, n(%) |  |  | <0.001 |  |  |  | 0.704 |
| Mild | 370 ( 8.8) | 67 ( 6.2) |  |  | 65 ( 6.3) | 63 ( 6.1) |  |
| Moderate | 3101 (73.8) | 705 (65.0) |  |  | 665 (64.2) | 683 (65.9) |  |
| Severe | 730 (17.4) | 312 (28.8) |  |  | 306 (29.5) | 290 (28.0) |  |
| Vaccination doses (%) |  |  | 0.331 |  |  |  | 0.995 |
| Unvaccinated | 1137 (27.1) | 327 (30.2) |  |  | 309 (29.8) | 313 (30.2) |  |
| One dose | 192 ( 4.6) | 55 ( 5.1) |  |  | 50 ( 4.8) | 52 ( 5.0) |  |
| Two doses | 669 (15.9) | 162 (14.9) |  |  | 159 (15.3) | 154 (14.9) |  |
| Three doses | 2171 (51.7) | 530 (48.9) |  |  | 510 (49.2) | 507 (48.9) |  |
| Four doses | 30 ( 0.7) | 9 ( 0.8) |  |  | 7 ( 0.7) | 9 ( 0.9) |  |
| Five doses | 2 ( 0.0) | 1 ( 0.1) |  |  | 1 ( 0.1) | 1 ( 0.1) |  |
| Concomitant systemic steroid, n(%) |  |  | <0.001 |  |  |  | 0.327 |
| No | 3165 (75.3) | 596 (55.0) |  |  | 614 (59.3) | 591 (57.0) |  |
| Yes | 1036 (24.7) | 488 (45.0) |  |  | 422 (40.7) | 445 (43.0) |  |
| Antibiotics, n(%) |  |  | <0.001 |  |  |  | 1 |
| No | 2801 (66.7) | 521 (48.1) |  |  | 511 (49.3) | 511 (49.3) |  |
| Yes | 1400 (33.3) | 563 (51.9) |  |  | 525 (50.7) | 525 (50.7) |  |
| Comorbidities, n(%) |  |  |  |  |  |  |  |
| Diabetes |  |  | 0.861 |  |  |  | 0.487 |
| No | 3357 (79.9) | 863 (79.6) |  |  | 806 (77.8) | 820 (79.2) |  |
| Yes | 844 (20.1) | 221 (20.4) |  |  | 230 (22.2) | 216 (20.8) |  |
| Hypertension |  |  | <0.001 |  |  |  | 0.724 |
| No | 2809 (66.9) | 585 (54.0) |  |  | 559 (54.0) | 568 (54.8) |  |
| Yes | 1392 (33.1) | 499 (46.0) |  |  | 477 (46.0) | 468 (45.2) |  |
| Cardio-cerebral diseases |  |  | <0.001 |  |  |  | 0.661 |
| No | 2818 (67.1) | 792 (73.1) |  |  | 736 (71.0) | 746 (72.0) |  |
| Yes | 1383 (32.9) | 292 (26.9) |  |  | 300 (29.0) | 290 (28.0) |  |
| Kidney diseases |  |  | <0.001 |  |  |  | 0.497 |
| No | 3438 (81.8) | 642 (59.2) |  |  | 652 (62.9) | 636 (61.4) |  |
| Yes | 763 (18.2) | 442 (40.8) |  |  | 384 (37.1) | 400 (38.6) |  |
| Primary malignant tumor |  |  | <0.001 |  |  |  | 0.789 |
| No | 2877 (68.5) | 959 (88.5) |  |  | 906 (87.5) | 911 (87.9) |  |
| Yes | 1324 (31.5) | 125 (11.5) |  |  | 130 (12.5) | 125 (12.1) |  |
| Chronic respiratory diseases, n(%) |  |  | 0.062 |  |  |  | 0.735 |
| No | 3491 (83.1) | 874 (80.6) |  |  | 846 (81.7) | 839 (81.0) |  |
| Yes | 710 (16.9) | 210 (19.4) |  |  | 190 (18.3) | 197 (19.0) |  |
| Autoimmune diseases, n(%) |  |  | 0.541 |  |  |  | 0.187 |
| No | 4010 (95.5) | 1040 (95.9) |  |  | 1006 (97.1) | 994 (95.9) |  |
| Yes | 191 ( 4.5) | 44 ( 4.1) |  |  | 30 ( 2.9) | 42 ( 4.1) |  |
| Laboratory parameters, (mean ± SD) |  |  |  |  |  |  |  |
| Neutrophil, ×10^9^/L | 5.79 (4.30) | 5.97 (3.71) | 0.194 |  | 6.21 (4.25) | 5.98 (3.69) | 0.194 |
| Lymphocyte, ×10^9^/L | 1.24 (2.15) | 1.06 (1.22) | 0.008 |  | 1.10 (0.86) | 1.08 (1.24) | 0.649 |
| Glucose, mmol/L | 7.16 (3.27) | 7.88 (3.37) | <0.001 |  | 7.87 (4.00) | 7.87 (3.40) | 0.998 |
| High-density lipoprotein, mmol/L | 1.06 (1.15) | 1.12 (1.65) | 0.187 |  | 1.13 (1.87) | 1.12 (1.69) | 0.893 |
| Low-density lipoprotein, mmol/L | 2.34 (1.31) | 2.37 (1.74) | 0.559 |  | 2.38 (1.92) | 2.38 (1.77) | 1 |
| Alanine aminotransferase, IU/L | 63.33 (194.86) | 56.47 (97.24) | 0.262 |  | 58.52 (128.15) | 56.60 (98.85) | 0.703 |
| Aspartate aminotransferase, IU/L | 83.11 (349.91) | 58.45 (88.24) | 0.021 |  | 58.04 (108.35) | 58.49 (90.01) | 0.918 |
| Creatinine, µmol/L | 116.66 (316.66) | 102.23 (179.64) | 0.149 |  | 111.10 (166.44) | 103.34 (183.59) | 0.314 |
| glomerular filtration rate, ml/min | 117.90 (121.44) | 106.05 (36.97) | 0.001 |  | 105.16 (65.60) | 105.64 (37.75) | 0.838 |
| C–reactive protein, mg/L | 51.20 (48.92) | 56.74 (56.07) | 0.001 |  | 60.17 (61.89) | 56.44 (55.37) | 0.149 |
| Procalcitonin, ng/ml | 2.23 (8.43) | 1.28 (4.04) | <0.001 |  | 1.37 (2.97) | 1.29 (4.13) | 0.593 |
| Prothrombin time, s | 15.11 (7.29) | 18.59 (9.30) | <0.001 |  | 17.61 (10.34) | 18.00 (8.88) | 0.348 |
| Activated partial thromboplastin time, s | 29.38 (7.92) | 25.23 (9.69) | <0.001 |  | 26.47 (8.43) | 25.72 (9.55) | 0.059 |
| Cholesterol, mmol/L | 4.15 (3.28) | 4.10 (1.76) | 0.623 |  | 4.14 (2.22) | 4.10 (1.79) | 0.73 |
| Triglyceride, mmol/L | 1.73 (3.41) | 1.61 (1.73) | 0.246 |  | 1.66 (2.29) | 1.63 (1.76) | 0.744 |
| Alkaline phosphatase, IU/L | 116.59 (118.53) | 96.90 (71.09) | <0.001 |  | 97.41 (57.49) | 97.63 (71.85) | 0.94 |
| Gamma-glutamyl transpeptidase, IU/L | 92.53 (136.87) | 88.71 (201.33) | 0.462 |  | 85.85 (119.32) | 84.04 (114.55) | 0.725 |
| Albumin, g/L | 36.06 (10.21) | 34.52 (12.32) | <0.001 |  | 34.64 (10.15) | 34.64 (12.53) | 0.988 |
| Total bilirubin, umol/L | 24.64 (51.78) | 15.04 (14.45) | <0.001 |  | 16.06 (18.11) | 15.17 (14.72) | 0.222 |

**Abbreviations:** BMI, Body mass index.

Table S3 Baseline characteristics of the population in the sensitivity analysis where patients who discharged within one day after receiving azvudine were excluded.

| Baseline characteristics | Before matching | | |  | After propensity score matching (1:1) | | |
| --- | --- | --- | --- | --- | --- | --- | --- |
|  | Control  （n=4201） | Azvudine  (n=1084) | *P* value |  | Control  （n=1022） | Azvudine  (n=1022) | *P* value |
| Sociodemographic information |  |  |  |  |  |  |  |
| Age, years (mean ± SD) | 62.57 (14.52) | 66.52 (14.34) | <0.001 |  | 66.74 (14.75) | 66.00 (14.22) | 0.253 |
| Gender, n(%) |  |  | 0.013 |  |  |  | 0.64 |
| Male | 2560 (61.1) | 707 (65.3) |  |  | 659 (65.6) | 648 (64.5) |  |
| Female | 1630 (38.9) | 376 (34.7) |  |  | 345 (34.4) | 356 (35.5) |  |
| BMI, kg/m^2^ (mean ± SD) | 24.16 (4.01) | 24.47 (4.22) | 0.028 |  | 24.38 (4.05) | 24.45 (4.23) | 0.678 |
| Severity at admission, n(%) |  |  | <0.001 |  |  |  | 0.547 |
| Mild | 370 ( 8.8) | 67 ( 6.2) |  |  | 66 ( 6.6) | 64 ( 6.4) |  |
| Moderate | 3094 (73.8) | 704 (65.0) |  |  | 641 (63.8) | 664 (66.1) |  |
| Severe | 726 (17.3) | 312 (28.8) |  |  | 297 (29.6) | 276 (27.5) |  |
| Vaccination doses (%) |  |  | 0.334 |  |  |  | 0.816 |
| Unvaccinated | 1135 (27.1) | 327 (30.2) |  |  | 317 (31.6) | 301 (30.0) |  |
| One dose | 192 ( 4.6) | 55 ( 5.1) |  |  | 57 ( 5.7) | 51 ( 5.1) |  |
| Two doses | 667 (15.9) | 162 (15.0) |  |  | 152 (15.1) | 151 (15.0) |  |
| Three doses | 2164 (51.6) | 529 (48.8) |  |  | 471 (46.9) | 492 (49.0) |  |
| Four doses | 30 ( 0.7) | 9 ( 0.8) |  |  | 5 ( 0.5) | 8 ( 0.8) |  |
| Five doses | 2 ( 0.0) | 1 ( 0.1) |  |  | 2 ( 0.2) | 1 ( 0.1) |  |
| Concomitant systemic steroid, n(%) |  |  | <0.001 |  |  |  | 0.586 |
| No | 3159 (75.4) | 595 (54.9) |  |  | 602 (60.0) | 589 (58.7) |  |
| Yes | 1031 (24.6) | 488 (45.1) |  |  | 402 (40.0) | 415 (41.3) |  |
| Antibiotics, n(%) |  |  | <0.001 |  |  |  | 0.532 |
| No | 2792 (66.6) | 520 (48.0) |  |  | 496 (49.4) | 511 (50.9) |  |
| Yes | 1398 (33.4) | 563 (52.0) |  |  | 508 (50.6) | 493 (49.1) |  |
| Comorbidities, n(%) |  |  |  |  |  |  |  |
| Diabetes |  |  | 0.853 |  |  |  | 0.484 |
| No | 3348 (79.9) | 862 (79.6) |  |  | 776 (77.3) | 790 (78.7) |  |
| Yes | 842 (20.1) | 221 (20.4) |  |  | 228 (22.7) | 214 (21.3) |  |
| Hypertension |  |  | <0.001 |  |  |  | 0.501 |
| No | 2800 (66.8) | 585 (54.0) |  |  | 545 (54.3) | 561 (55.9) |  |
| Yes | 1390 (33.2) | 498 (46.0) |  |  | 459 (45.7) | 443 (44.1) |  |
| Cardio-cerebral diseases |  |  | <0.001 |  |  |  | 0.054 |
| No | 2809 (67.0) | 791 (73.0) |  |  | 672 (66.9) | 713 (71.0) |  |
| Yes | 1381 (33.0) | 292 (27.0) |  |  | 332 (33.1) | 291 (29.0) |  |
| Kidney diseases |  |  | <0.001 |  |  |  | 0.644 |
| No | 3431 (81.9) | 642 (59.3) |  |  | 629 (62.6) | 640 (63.7) |  |
| Yes | 759 (18.1) | 441 (40.7) |  |  | 375 (37.4) | 364 (36.3) |  |
| Primary malignant tumor |  |  | <0.001 |  |  |  | 0.892 |
| No | 2869 (68.5) | 958 (88.5) |  |  | 882 (87.8) | 879 (87.5) |  |
| Yes | 1321 (31.5) | 125 (11.5) |  |  | 122 (12.2) | 125 (12.5) |  |
| Chronic respiratory diseases, n(%) |  |  | 0.062 |  |  |  | 0.57 |
| No | 3481 (83.1) | 873 (80.6) |  |  | 807 (80.4) | 818 (81.5) |  |
| Yes | 709 (16.9) | 210 (19.4) |  |  | 197 (19.6) | 186 (18.5) |  |
| Autoimmune diseases, n(%) |  |  | 0.534 |  |  |  | 0.646 |
| No | 3999 (95.4) | 1039 (95.9) |  |  | 967 (96.3) | 962 (95.8) |  |
| Yes | 191 ( 4.6) | 44 ( 4.1) |  |  | 37 ( 3.7) | 42 ( 4.2) |  |
| Laboratory parameters, (mean ± SD) |  |  |  |  |  |  |  |
| Neutrophil, ×10^9^/L | 5.79 (4.64) | 6.00 (4.04) | 0.178 |  | 6.21 (4.69) | 5.96 (4.04) | 0.187 |
| Lymphocyte, ×10^9^/L | 1.25 (2.52) | 1.05 (1.24) | 0.012 |  | 1.06 (0.80) | 1.08 (1.28) | 0.766 |
| Glucose, mmol/L | 7.17 (3.85) | 7.99 (4.13) | <0.001 |  | 7.99 (4.73) | 7.87 (3.96) | 0.547 |
| High-density lipoprotein, mmol/L | 1.06 (1.61) | 1.08 (1.68) | 0.8 |  | 1.10 (1.90) | 1.08 (1.74) | 0.876 |
| Low-density lipoprotein, mmol/L | 2.35 (1.79) | 2.41 (2.47) | 0.331 |  | 2.41 (2.66) | 2.42 (2.55) | 0.953 |
| Alanine aminotransferase, IU/L | 65.19 (221.70) | 56.00 (102.45) | 0.185 |  | 61.73 (152.89) | 55.96 (103.25) | 0.322 |
| Aspartate aminotransferase, IU/L | 81.38 (358.99) | 58.44 (113.50) | 0.038 |  | 74.04 (294.75) | 58.42 (117.12) | 0.119 |
| Creatinine, µmol/L | 120.28 (414.74) | 100.07 (187.44) | 0.118 |  | 106.12 (128.44) | 101.92 (194.37) | 0.568 |
| glomerular filtration rate, ml/min | 117.76 (166.03) | 96.27 (103.90) | <0.001 |  | 91.23 (85.99) | 96.79 (107.61) | 0.201 |
| C–reactive protein, mg/L | 51.42 (67.89) | 56.80 (71.13) | 0.021 |  | 59.03 (74.90) | 56.93 (72.07) | 0.522 |
| Procalcitonin, ng/ml | 2.11 (10.78) | 1.15 (6.68) | 0.005 |  | 1.33 (5.86) | 1.21 (6.93) | 0.674 |
| Prothrombin time, s | 14.89 (8.12) | 18.69 (10.31) | <0.001 |  | 17.61 (9.72) | 17.67 (9.51) | 0.884 |
| Activated partial thromboplastin time, s | 29.33 (9.54) | 25.23 (10.91) | <0.001 |  | 26.73 (10.11) | 26.10 (10.75) | 0.176 |
| Cholesterol, mmol/L | 4.10 (3.56) | 4.02 (1.94) | 0.488 |  | 3.98 (2.13) | 4.04 (1.99) | 0.501 |
| Triglyceride, mmol/L | 1.76 (4.79) | 1.57 (2.82) | 0.214 |  | 1.56 (2.11) | 1.60 (2.92) | 0.714 |
| Alkaline phosphatase, IU/L | 116.29 (127.26) | 97.34 (75.43) | <0.001 |  | 102.06 (91.80) | 98.99 (77.74) | 0.419 |
| Gamma-glutamyl transpeptidase, IU/L | 93.92 (175.90) | 89.62 (214.04) | 0.493 |  | 90.49 (221.42) | 90.62 (219.99) | 0.99 |
| Albumin, g/L | 36.13 (11.54) | 34.67 (12.91) | <0.001 |  | 34.98 (10.77) | 34.96 (13.30) | 0.959 |
| Total bilirubin, umol/L | 24.78 (55.86) | 14.94 (26.13) | <0.001 |  | 15.59 (19.61) | 15.01 (27.05) | 0.58 |

**Abbreviations:** BMI, Body mass index.

Table S4 Baseline characteristics of the study population collected from the First Affiliated Hospital of Henan University of Science & Technology

| Baseline characteristics | Before matching | | |  | After propensity score matching (1:1) | | |
| --- | --- | --- | --- | --- | --- | --- | --- |
|  | Control  （n=387） | Azvudine  (n=271) | *P* value |  | Control  （n=181） | Azvudine  (n=181) | *P* value |
| Sociodemographic information |  |  |  |  |  |  |  |
| Age, years (mean ± SD) | 65.49 (14.94) | 70.88 (14.30) | <0.001 |  | 68.59 (14.82) | 69.41 (14.53) | 0.594 |
| Gender, n(%) |  |  | 0.088 |  |  |  | 1 |
| Male | 229 (59.2) | 179 (66.1) |  |  | 113 (62.4) | 112 (61.9) |  |
| Female | 158 (40.8) | 92 (33.9) |  |  | 68 (37.6) | 69 (38.1) |  |
| Severity at admission, n(%) |  |  | <0.001 |  |  |  | 0.699 |
| Mild | 241 (62.3) | 96 (35.4) |  |  | 85 (47.0) | 78 (43.1) |  |
| Moderate | 104 (26.9) | 100 (36.9) |  |  | 66 (36.5) | 68 (37.6) |  |
| Severe | 42 (10.9) | 75 (27.7) |  |  | 30 (16.6) | 35 (19.3) |  |
| Vaccination doses (%) |  |  | <0.001 |  |  |  | 0.541 |
| Unvaccinated | 102 (26.4) | 113 (41.7) |  |  | 62 (34.3) | 69 (38.1) |  |
| One dose | 13 ( 3.4) | 16 ( 5.9) |  |  | 7 ( 3.9) | 7 ( 3.9) |  |
| Two doses | 61 (15.8) | 30 (11.1) |  |  | 28 (15.5) | 22 (12.2) |  |
| Three doses | 209 (54.0) | 110 (40.6) |  |  | 82 (45.3) | 83 (45.9) |  |
| Four doses | 2 ( 0.5) | 2 ( 0.7) |  |  | 2 ( 1.1) | 0 ( 0.0) |  |
| Concomitant systemic steroid, n(%) |  |  | <0.001 |  |  |  | 0.293 |
| No | 256 (66.1) | 112 (41.3) |  |  | 95 (52.5) | 84 (46.4) |  |
| Yes | 131 (33.9) | 159 (58.7) |  |  | 86 (47.5) | 97 (53.6) |  |
| Antibiotics, n(%) |  |  | <0.001 |  |  |  | 0.45 |
| No | 196 (50.6) | 93 (34.3) |  |  | 74 (40.9) | 66 (36.5) |  |
| Yes | 191 (49.4) | 178 (65.7) |  |  | 107 (59.1) | 115 (63.5) |  |
| Comorbidities, n(%) |  |  |  |  |  |  |  |
| Diabetes |  |  | 0.203 |  |  |  | 0.349 |
| No | 298 (77.0) | 196 (72.3) |  |  | 135 (74.6) | 126 (69.6) |  |
| Yes | 89 (23.0) | 75 (27.7) |  |  | 46 (25.4) | 55 (30.4) |  |
| Hypertension |  |  | 0.395 |  |  |  | 0.518 |
| No | 241 (62.3) | 159 (58.7) |  |  | 114 (63.0) | 107 (59.1) |  |
| Yes | 146 (37.7) | 112 (41.3) |  |  | 67 (37.0) | 74 (40.9) |  |
| Cardio-cerebral diseases |  |  | 0.158 |  |  |  | 0.915 |
| No | 174 (45.0) | 106 (39.1) |  |  | 79 (43.6) | 77 (42.5) |  |
| Yes | 213 (55.0) | 165 (60.9) |  |  | 102 (56.4) | 104 (57.5) |  |
| Kidney diseases |  |  | 0.205 |  |  |  | 0.647 |
| No | 283 (73.1) | 185 (68.3) |  |  | 124 (68.5) | 129 (71.3) |  |
| Yes | 104 (26.9) | 86 (31.7) |  |  | 57 (31.5) | 52 (28.7) |  |
| Primary malignant tumor |  |  | <0.001 |  |  |  | 0.62 |
| No | 245 (63.3) | 219 (80.8) |  |  | 136 (75.1) | 141 (77.9) |  |
| Yes | 142 (36.7) | 52 (19.2) |  |  | 45 (24.9) | 40 (22.1) |  |
| Chronic respiratory diseases, n(%) |  |  | 0.025 |  |  |  | 0.773 |
| No | 305 (78.8) | 233 (86.0) |  |  | 151 (83.4) | 154 (85.1) |  |
| Yes | 82 (21.2) | 38 (14.0) |  |  | 30 (16.6) | 27 (14.9) |  |
| Autoimmune diseases, n(%) |  |  | 0.319 |  |  |  | 0.798 |
| No | 355 (91.7) | 255 (94.1) |  |  | 172 (95.0) | 174 (96.1) |  |
| Yes | 32 ( 8.3) | 16 ( 5.9) |  |  | 9 ( 5.0) | 7 ( 3.9) |  |
| Laboratory parameters, (mean ± SD) |  |  |  |  |  |  |  |
| Neutrophil, ×10^9^/L | 5.57 (4.97) | 6.12 (4.28) | 0.142 |  | 5.67 (4.69) | 5.81 (4.17) | 0.762 |
| Lymphocyte, ×10^9^/L | 1.21 (1.16) | 0.90 (0.62) | <0.001 |  | 1.01 (0.58) | 0.95 (0.68) | 0.366 |
| Glucose, mmol/L | 7.39 (4.07) | 8.66 (4.38) | <0.001 |  | 7.72 (3.97) | 8.21 (4.15) | 0.25 |
| High-density lipoprotein, mmol/L | 1.04 (0.40) | 1.02 (0.37) | 0.437 |  | 1.07 (0.40) | 1.05 (0.37) | 0.587 |
| Low-density lipoprotein, mmol/L | 2.15 (0.86) | 2.06 (0.81) | 0.163 |  | 2.13 (0.89) | 2.13 (0.90) | 0.991 |
| Alanine aminotransferase, IU/L | 55.70 (89.92) | 55.56 (108.43) | 0.985 |  | 49.56 (58.35) | 58.93 (129.39) | 0.375 |
| Aspartate aminotransferase, IU/L | 65.39 (149.60) | 51.64 (52.83) | 0.147 |  | 48.29 (61.68) | 52.39 (57.85) | 0.515 |
| Creatinine, µmol/L | 88.67 (109.16) | 98.77 (118.54) | 0.26 |  | 95.18 (101.61) | 95.01 (120.23) | 0.988 |
| C–reactive protein, mg/L | 55.39 (61.59) | 51.02 (56.01) | 0.353 |  | 54.40 (55.47) | 53.61 (54.71) | 0.891 |
| Procalcitonin, ng/ml | 2.59 (9.28) | 2.57 (9.13) | 0.975 |  | 2.05 (6.19) | 2.22 (8.28) | 0.824 |
| Prothrombin time, s | 13.04 (4.29) | 13.02 (3.53) | 0.97 |  | 12.63 (2.43) | 12.62 (2.68) | 0.957 |
| Activated partial thromboplastin time, s | 35.09 (9.36) | 36.76 (9.47) | 0.026 |  | 35.87 (9.21) | 35.72 (8.11) | 0.874 |
| Cholesterol, mmol/L | 3.84 (1.33) | 3.67 (1.13) | 0.079 |  | 3.71 (1.35) | 3.69 (1.10) | 0.884 |
| Triglyceride, mmol/L | 1.65 (2.08) | 1.37 (0.85) | 0.034 |  | 1.30 (0.78) | 1.29 (0.66) | 0.868 |
| Alkaline phosphatase, IU/L | 110.99 (103.60) | 88.20 (49.09) | 0.001 |  | 90.48 (45.59) | 90.35 (52.71) | 0.98 |
| Gamma-glutamyl transpeptidase, IU/L | 99.09 (176.20) | 70.65 (85.53) | 0.014 |  | 73.27 (93.43) | 76.87 (104.31) | 0.729 |
| Albumin, g/L | 36.25 (6.18) | 34.79 (5.72) | 0.002 |  | 36.11 (6.01) | 35.59 (5.45) | 0.389 |
| Total bilirubin, umol/L | 22.59 (47.70) | 15.31 (22.58) | 0.02 |  | 14.29 (8.49) | 14.40 (11.04) | 0.913 |

**Abbreviations:** BMI, Body mass index.

Table S5 Baseline characteristics of the study population coinfected with SARS-CoV-2 and HBV/HCV

| Baseline characteristics | Before matching | | |  | After propensity score matching (2:1) | | |
| --- | --- | --- | --- | --- | --- | --- | --- |
|  | Control  （n=285） | Azvudine  (n=55) | *P* value |  | Control  （n=110） | Azvudine  (n=55) | *P* value |
| Sociodemographic information |  |  |  |  |  |  |  |
| Age, years (mean ± SD) | 58.42 (12.74) | 59.29 (14.51) | 0.652 |  | 58.28 (14.52) | 59.29 (14.51) | 0.674 |
| Gender, n(%) |  |  | 0.768 |  |  |  | 0.293 |
| Male | 167 (58.6) | 34 (61.8) |  |  | 57 (51.8) | 34 (61.8) |  |
| Female | 118 (41.4) | 21 (38.2) |  |  | 53 (48.2) | 21 (38.2) |  |
| BMI (mean ± SD) | 23.85 (4.08) | 23.72 (3.56) | 0.824 |  | 24.38 (4.32) | 23.72 (3.56) | 0.328 |
| Type of hepatitis, n(%) |  |  | 0.976 |  |  |  | 0.823 |
| HBV | 240 (84.2) | 47 (85.5) |  |  | 91 (82.7) | 47 (85.5) |  |
| HCV | 45 (15.8) | 8 (14.5) |  |  | 19 (17.3) | 8 (14.5) |  |
| Severity at admission, n(%) |  |  | 0.006 |  |  |  | 0.553 |
| Mild | 31 (10.9) | 5 ( 9.1) |  |  | 11 (10.0) | 5 ( 9.1) |  |
| Moderate | 226 (79.3) | 36 (65.5) |  |  | 79 (71.8) | 36 (65.5) |  |
| Severe | 28 ( 9.8) | 14 (25.5) |  |  | 20 (18.2) | 14 (25.5) |  |
| Vaccination doses (%) |  |  | 0.008 |  |  |  | 0.605 |
| Unvaccinated | 99 (34.7) | 28 (50.9) |  |  | 53 (48.2) | 28 (50.9) |  |
| One dose | 7 ( 2.5) | 5 ( 9.1) |  |  | 5 ( 4.5) | 5 ( 9.1) |  |
| Two doses | 57 (20.0) | 5 ( 9.1) |  |  | 10 ( 9.1) | 5 ( 9.1) |  |
| Three doses | 121 (42.5) | 17 (30.9) |  |  | 42 (38.2) | 17 (30.9) |  |
| Four doses | 1 ( 0.4) | 0 ( 0.0) |  |  | 0 ( 0.0) | 0 ( 0.0) |  |
| Concomitant systemic steroid, n(%) |  |  | 0.304 |  |  |  | 1 |
| No | 209 (73.3) | 36 (65.5) |  |  | 73 (66.4) | 36 (65.5) |  |
| Yes | 76 (26.7) | 19 (34.5) |  |  | 37 (33.6) | 19 (34.5) |  |
| Antibiotics, n(%) |  |  | <0.001 |  |  |  | 0.403 |
| No | 221 (77.5) | 29 (52.7) |  |  | 67 (60.9) | 29 (52.7) |  |
| Yes | 64 (22.5) | 26 (47.3) |  |  | 43 (39.1) | 26 (47.3) |  |
| Comorbidities, n(%) |  |  |  |  |  |  |  |
| Diabetes |  |  | 0.367 |  |  |  | 1 |
| No | 226 (79.3) | 40 (72.7) |  |  | 80 (72.7) | 40 (72.7) |  |
| Yes | 59 (20.7) | 15 (27.3) |  |  | 30 (27.3) | 15 (27.3) |  |
| Hypertension |  |  | 0.119 |  |  |  | 0.698 |
| No | 185 (64.9) | 29 (52.7) |  |  | 63 (57.3) | 29 (52.7) |  |
| Yes | 100 (35.1) | 26 (47.3) |  |  | 47 (42.7) | 26 (47.3) |  |
| Cardio-cerebral diseases |  |  | 0.631 |  |  |  | 1 |
| No | 209 (73.3) | 38 (69.1) |  |  | 76 (69.1) | 38 (69.1) |  |
| Yes | 76 (26.7) | 17 (30.9) |  |  | 34 (30.9) | 17 (30.9) |  |
| Kidney diseases |  |  | 0.003 |  |  |  | 0.448 |
| No | 231 (81.1) | 34 (61.8) |  |  | 76 (69.1) | 34 (61.8) |  |
| Yes | 54 (18.9) | 21 (38.2) |  |  | 34 (30.9) | 21 (38.2) |  |
| Primary malignant tumor |  |  | <0.001 |  |  |  | 0.521 |
| No | 163 (57.2) | 47 (85.5) |  |  | 88 (80.0) | 47 (85.5) |  |
| Yes | 122 (42.8) | 8 (14.5) |  |  | 22 (20.0) | 8 (14.5) |  |
| Chronic respiratory diseases, n(%) |  |  | 0.2 |  |  |  | 0.815 |
| No | 258 (90.5) | 46 (83.6) |  |  | 95 (86.4) | 46 (83.6) |  |
| Yes | 27 ( 9.5) | 9 (16.4) |  |  | 15 (13.6) | 9 (16.4) |  |
| Autoimmune diseases, n(%) |  |  | 0.958 |  |  |  | 1 |
| No | 273 (95.8) | 52 (94.5) |  |  | 105 (95.5) | 52 (94.5) |  |
| Yes | 12 ( 4.2) | 3 ( 5.5) |  |  | 5 ( 4.5) | 3 ( 5.5) |  |
| Laboratory parameters, (mean ± SD) |  |  |  |  |  |  |  |
| Neutrophil, ×10^9^/L | 4.40 (3.52) | 4.55 (4.86) | 0.784 |  | 4.52 (2.86) | 4.55 (4.86) | 0.963 |
| Lymphocyte, ×10^9^/L | 1.13 (1.31) | 0.95 (0.64) | 0.316 |  | 1.00 (0.53) | 0.95 (0.64) | 0.566 |
| Glucose, mmol/L | 6.86 (4.48) | 7.29 (4.88) | 0.52 |  | 6.89 (3.92) | 7.29 (4.88) | 0.575 |
| High-density lipoprotein, mmol/L | 1.31 (3.41) | 1.64 (5.02) | 0.555 |  | 1.40 (3.88) | 1.64 (5.02) | 0.74 |
| Low-density lipoprotein, mmol/L | 2.67 (4.65) | 2.90 (4.95) | 0.736 |  | 2.86 (5.16) | 2.90 (4.95) | 0.962 |
| Alanine aminotransferase, IU/L | 32.70 (37.37) | 33.24 (30.86) | 0.92 |  | 33.96 (36.74) | 33.24 (30.86) | 0.9 |
| Aspartate aminotransferase, IU/L | 38.24 (36.43) | 42.72 (37.89) | 0.407 |  | 38.63 (44.64) | 42.72 (37.89) | 0.561 |
| Creatinine, µmol/L | 213.31 (1014.69) | 234.22 (629.51) | 0.883 |  | 185.81 (384.92) | 234.22 (629.51) | 0.542 |
| glomerular filtration rate, ml/min | 102.80 (135.59) | 99.40 (135.12) | 0.865 |  | 112.17 (188.06) | 99.40 (135.12) | 0.654 |
| C–reactive protein, mg/L | 34.17 (61.71) | 33.48 (38.91) | 0.937 |  | 32.77 (44.56) | 33.48 (38.91) | 0.92 |
| Procalcitonin, ng/ml | 0.75 (3.28) | 0.74 (2.42) | 0.984 |  | 0.55 (3.03) | 0.74 (2.42) | 0.688 |
| Prothrombin time, s | 13.54 (5.41) | 16.40 (10.54) | 0.003 |  | 14.02 (7.29) | 16.40 (10.54) | 0.091 |
| Activated partial thromboplastin time, s | 30.83 (6.87) | 29.09 (11.62) | 0.131 |  | 31.24 (8.47) | 29.09 (11.62) | 0.177 |
| Cholesterol, mmol/L | 3.91 (3.28) | 4.27 (4.78) | 0.494 |  | 4.22 (3.87) | 4.27 (4.78) | 0.934 |
| Triglyceride, mmol/L | 1.36 (2.49) | 1.94 (5.01) | 0.195 |  | 1.66 (3.90) | 1.94 (5.01) | 0.689 |
| Alkaline phosphatase, IU/L | 105.36 (82.81) | 83.43 (43.62) | 0.057 |  | 82.43 (37.95) | 83.43 (43.62) | 0.88 |
| Gamma-glutamyl transpeptidase, IU/L | 71.98 (129.85) | 59.12 (85.54) | 0.481 |  | 58.38 (65.95) | 59.12 (85.54) | 0.951 |
| Albumin, g/L | 35.87 (10.88) | 33.86 (12.91) | 0.226 |  | 34.87 (10.60) | 33.86 (12.91) | 0.595 |
| Total bilirubin, umol/L | 21.10 (44.28) | 12.23 (12.66) | 0.142 |  | 11.78 (7.23) | 12.23 (12.66) | 0.771 |

**Abbreviations:** BMI, Body mass index.
